# A dual role of RBM42 in modulating splicing and translation of CDKN1A/p21 during DNA damage response

**DOI:** 10.1038/s41467-023-43495-6

**Published:** 2023-11-22

**Authors:** Bella M. Ben-Oz, Feras E. Machour, Marian Nicola, Amir Argoetti, Galia Polyak, Rawad Hanna, Oded Kleifeld, Yael Mandel-Gutfreund, Nabieh Ayoub

**Affiliations:** Department of Biology, Technion - Israel Institute of Technology, Haifa, 3200003 Israel

**Keywords:** DNA damage response, DNA damage and repair

## Abstract

p53-mediated cell cycle arrest during DNA damage is dependent on the induction of p21 protein, encoded by the CDKN1A gene. p21 inhibits cyclin-dependent kinases required for cell cycle progression to guarantee accurate repair of DNA lesions. Hence, fine-tuning of p21 levels is crucial to preserve genomic stability. Currently, the multilayered regulation of p21 levels during DNA damage is not fully understood. Herein, we identify the human RNA binding motif protein 42 (RBM42) as a regulator of p21 levels during DNA damage. Genome-wide transcriptome and interactome analysis reveals that RBM42 alters the expression of p53-regulated genes during DNA damage. Specifically, we demonstrate that RBM42 facilitates CDKN1A splicing by counteracting the splicing inhibitory effect of RBM4 protein. Unexpectedly, we also show that RBM42, underpins translation of various splicing targets, including CDKN1A. Concordantly, transcriptome-wide mapping of RBM42-RNA interactions using eCLIP further substantiates the dual function of RBM42 in regulating splicing and translation of its target genes, including CDKN1A. Collectively, our data show that RBM42 couples splicing and translation machineries to fine-tune gene expression during DNA damage response.

## Abstract (web summary)

Here the authors show a function of RBM42 in regulating splicing and translation of the p53-target gene CDKN1A/p21. This makes RBM42 an RNA-binding protein that links splicing and translation to fine-tune gene expression during DNA damage.

## Introduction

The gene TP53, encoding for p53 protein, is the most frequently mutated gene in human cancer. Over 50% of human cancers carry loss of function mutations in p53, and hence it is one of the extensively studied tumor suppressors in the field of cancer research. Under normal conditions, p53 protein is maintained at low levels, primarily since it is targeted for proteasomal degradation by the E3 ubiquitin ligase MDM2^1–4^. In response to DNA damage, p53 protein levels become substantially elevated and activate the expression of hundreds of genes that coordinate the cellular response to DNA damage, which are implicated in cell cycle arrest, DNA repair, senescence and apoptosis^2,3,5–8^.

Among the p53 transcriptional target genes is the cyclin-dependent kinase inhibitor 1A (CDKN1A) gene that produces pre-mRNA consisting of three exons and two introns encoding p21 protein. Upon DNA damage, p53 binds two consensus sequences near CDKN1A promoter and drives its transcription^9–11^. p21 protein interacts with and inhibits cyclin-CDK2, -CDK1, and -CDK4/6 complexes, thereby regulating cell cycle progression during DNA damage^10,12,13^. Furthermore, it was shown that p21 induces senescence and protects the cells from p53-mediated apoptosis following DNA damage^14,15^. Since p21 plays an important role in regulating cell cycle progression, its levels must be tightly controlled. Indeed, multi-layered regulatory mechanisms, involving mRNA stability, splicing, translation and proteolytic degradation, act concertedly to calibrate p21 expression levels^16–22^. For example, the splicing factors SKIP and U2AF65 regulate the splicing of CDKN1A pre-mRNA during DNA damage^16^. CELF6 and RBM24 regulate CDKN1A mRNA stability via binding to the 3´-untranslated region (3´-UTR) of CDKN1A transcript^19,21^. NSUN2 and METTL3/METTL14 complex regulates CDKN1A translation via promoting m5C and m6A methylation of CDKN1A mRNA, respectively^18^. GCN2 kinase underpins CDKN1A translation through phosphorylation of the eukaryotic translation initiation factor eIF2α^22^. Additionally, CUGBP1 and CRT proteins compete for binding to CDKN1A mRNA and regulate its translation^17^.

RNA binding motif protein 42 (RBM42) is an understudied gene mapped to 19q13.12 region and codes for a protein that consists of 480 amino acids, containing one RNA recognition motif (RRM) at its C-terminal region. RBM42 RRM domain consists of 72 amino acids harboring two main sequences. The first is ribonucleoprotein 1 (RNP 1), an octapeptide sequence consists of eight conserved amino acids that are mainly aromatic and positively charged, and the second is a ribonucleoprotein 2 (RNP 2) which contains six conserved amino acids. While previous report showed that RBM42 binds RNA in vitro^23^, the biological function of human RBM42 remains largely unknown. Interestingly, the toxoplasma gondii orthologue of human RBM42, *TgRRM1*, interacts with the spliceosome subcomplex U4/U6 and U5 small nuclear ribonucleoprotein particles (snRNPs) and regulates mRNA splicing^24^. Also, the fungus Fusarium graminearum ortholog of human RBM42, FgRbp1, regulates pre-mRNA splicing via interaction with U2AF23 splicing factor^25^. Recently, human RBM42 was identified as an integral component of the major spliceosome building block, U4/U6.U5 triple small nuclear ribonucleoprotein (tri-snRNP), and the pre-B complex, suggesting that it might be involved in RNA splicing^26–28^. Interestingly, a recent study showed that RBM42 bind to specific 5′UTR sequences and suppress translation of a subset of c-Myc target genes^29^.

Herein, we identify human RBM42 as a regulator of gene expression, including p53-target genes, during DNA damage. Specifically, we report a previously unrecognized dual role of RBM42 in regulating splicing and translation of CDKN1A RNA during DNA damage, and provide mechanistic insights into its activities. Collectively, our data provide an example for coordination between splicing and translation machineries mediated by the same RNA processing factor, RBM42. Such coordination is presumably critical for precise fine-tuning of gene expression during DNA damage response (DDR) to preserve genome stability.

## Results

### Human RBM42 alters the expression of p53-regulated genes during DNA damage

**Fig. 1:**
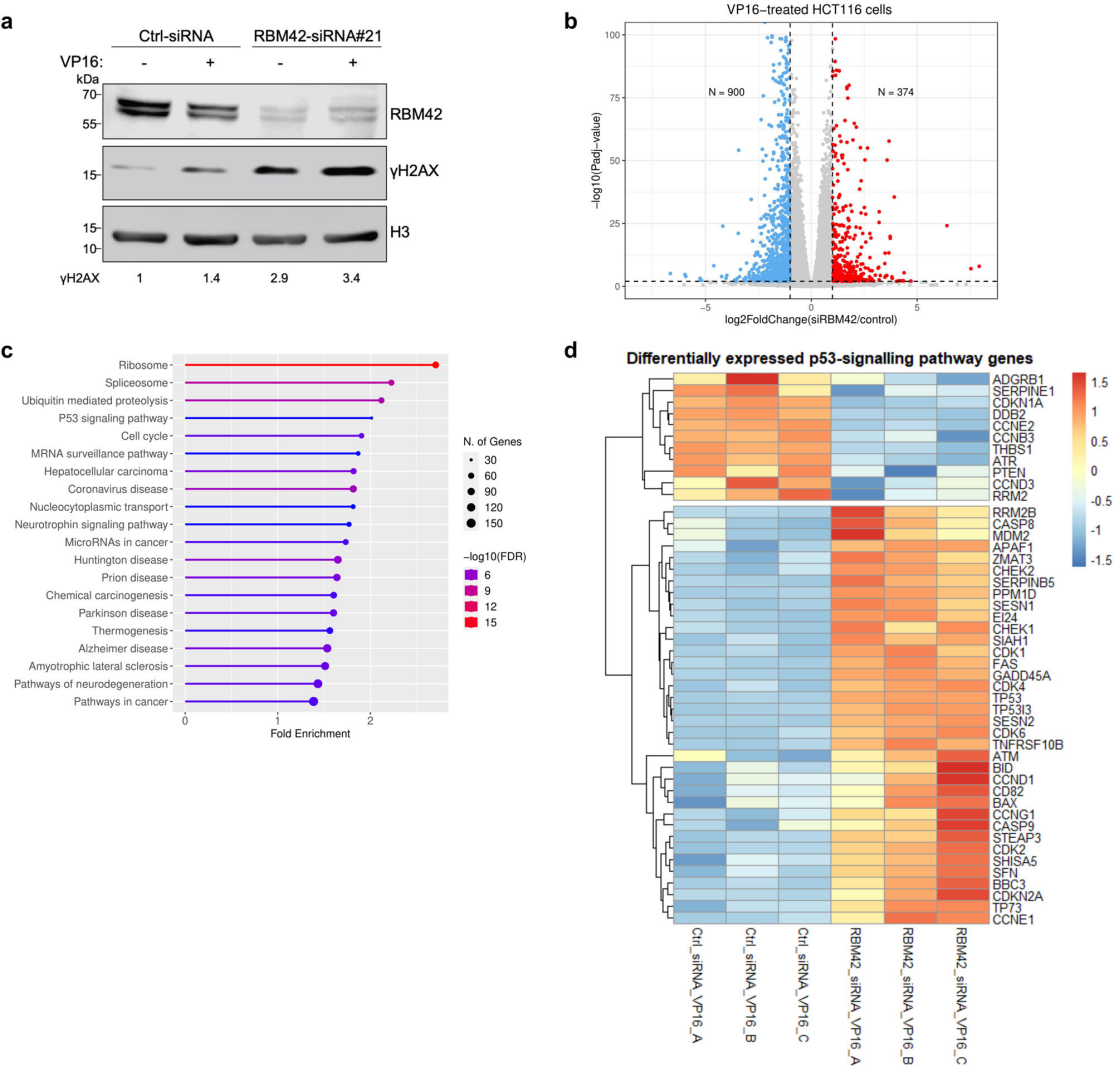
RBM42 regulates the expression of p53-related genes during DNA damage. **a** Western blot analysis shows RBM42-dependent increase in γH2AX levels before and after VP16 treatment. HCT116 cells transfected with control (Ctrl) or RBM42 siRNA were left untreated (UT) or treated with 20 μM VP16 for 12 h and subjected to hot-lysis protein extraction. Band intensities of γH2AX were normalized to the intensities of their respective H3 bands and are shown at the bottom of the blot. Protein molecular weight sizes are indicated at the left side of the western blots. **b** Volcano plot summarizing differential gene expression data obtained from RNA-seq analysis between control and siRBM42-transfected HCT116 cells treated with 20 μM VP16 for 12 h. Upregulated genes with log2FoldChange(siRBM42/control) > 1 and *P*adj-value < 0.01 are marked in red, while downregulated genes with log2FoldChange(siRBM42/control) < -1 and *P*adj-value < 0.01 are marked in blue. Padj is adjusted value calculated by Wald test statistic corrected for multiple testing. N indicates the number of significantly upregulated or downregulated genes. **c** KEGG pathway enrichment analysis of differentially expressed genes obtained from RNA-seq data between control and siRBM42-transfected HCT116 cells following VP16 treatment. **d** Heatmap summarizing the differentially expressed p53-signalling pathway genes in siRBM42-transfected HCT116 cells compared to control cells following VP16 treatment. The relative expression of p53-signalling pathway genes with |log2FoldChange(siRBM42/control)| > 1 and *P*adj-value < 0.01 is presented in each row. Source data are provided as a Source Data file.

We became interested in RBM42 since its depletion leads to an increase in γH2AX levels (Fig. 1a)^30^, suggesting that it might be implicated in DDR. To explore RBM42 role in DDR, we sought to determine the transcriptome of RBM42-proficient and -deficient cells during DNA damage. RNA samples were prepared from etoposide (VP16) treated and untreated HCT116 cells transfected with control or RBM42 siRNA, and subjected to deep RNA sequencing (RNA-seq) (Supplementary Fig. 1b-c). Of note, RBM42 transcriptome was determined using the more effective RBM42 siRNA sequence (siRNA#21), and subsequent validation was carried out using two different RBM42 siRNA sequences (siRNA#21&#22) (Supplementary Fig. 1a). Gene expression analysis revealed that RBM42 knockdown significantly alters the expression of 1325 and 1274 genes before and after VP16 treatment, respectively ( | fold-change(FC) | ≥ 2; *P*_adj_ < 0.01) (Fig. 1b; Supplementary Fig. 1d; Supplementary Data 1). Notably, KEGG pathway enrichment analysis revealed that RBM42-regulated genes are enriched in various pathways including ribosome, spliceosme, cell cycle and p53-signalling pathway (Fig. 1c; Supplementary Fig. 1e). Interestingly, while the levels of p53 and most of its target genes were increased in RBM42-deficient cells upon VP16 treatment, a smaller subset of p53-responsive genes, including CDKN1A, unexpectedly exhibited lower expression levels (Fig. 1d).

### RBM42 regulates p21 protein levels in a p53-independent manner during DNA damage

**Fig. 2:**
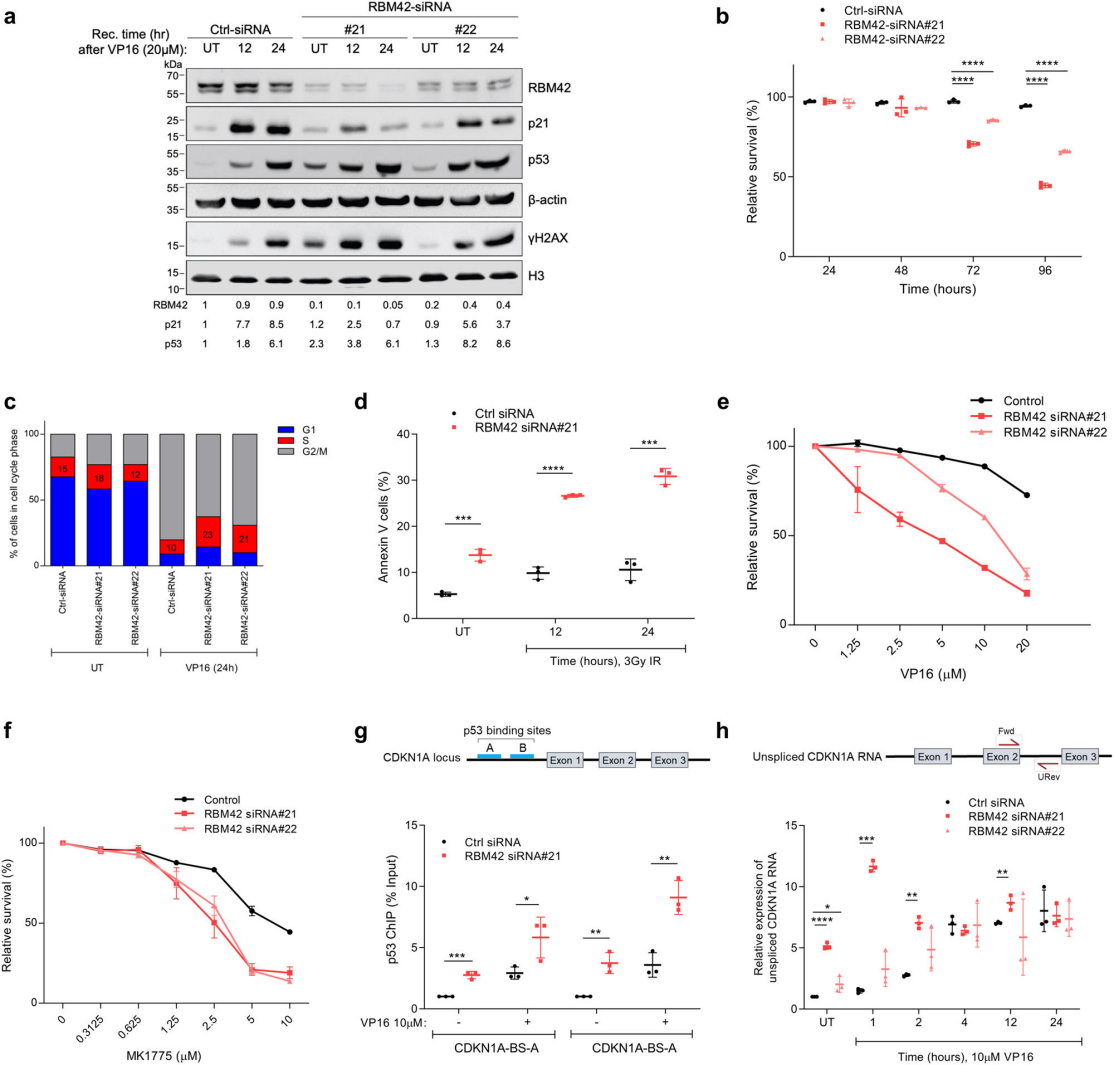
RBM42 regulates p21 protein levels in a p53-independent manner and phenocopies p21 deficiency during DNA damage. **a** Western blot analysis shows that RBM42 depletion impairs the DNA damage-induced increase of p21 protein levels. Band intensities were normalized to β-actin bands and are shown at the bottom of the blot. **b** RBM42 depletion decreases cell viability. HCT116 cells transfected with control (Ctrl) or RBM42 siRNA were stained with trypan-blue at the indicated time points and relative cell survival was determined. *P* value(si#21, 72 h) = 0.00001; *P* value(si#22, 72 h) = 0.00009; P-*value*(si#21, 96 h) = 0.00000007; P-*value*(si#22, 96 h) = 0.000002. **c** RBM42 depletion leads to defective G1\S arrest. Control and RBM42-deficient cells were treated with 10μM VP16 and samples were collected at the indicated time points for cell cycle analysis by flow cytometry. **d** Annexin-V assay shows that RBM42 deficiency increases apoptosis rate before and after DNA damage. Bar graph representing percentage of apoptotic cells (Annexin-V positive, PI negative) as measured by flow-cytometry. *P* value(UT) = 0.0004; *P* value(12 h) = 0.00003; P-*value*(24 h) = 0.0003. **e**, **f** Short-term cell viability assay in RBM42 -proficient and -deficient HCT116 cells treated with increasing concentrations of VP16 (e), and WEE1 inhibitor MK1775 (**f**). **g** p53 ChIP-qPCR shows that RBM42 knockdown leads to an increase in p53 protein levels at CDKN1A promoter before and after VP16. (Top) Schematic representation of two p53 binding sites (BS-A and BS-B) at CDKN1A promoter. *P* value(BS-A UT) = 0.0006; P-*value*(BS-A VP16) = 0.04; P-*value*(BS-B UT) = 0.005; P-*value*(BS-B VP16) = 0.005. **h** RBM42 depletion increases the levels of CDKN1A unspliced transcript during DNA damage. HCT116 cells were transfected with control or RBM42 siRNA and treated with VP16 or left untreated. RT-qPCR analysis was used to detect unspliced CDKN1A transcript. (Top) Schematic diagram shows the position of the PCR primers (arrows) used to amplify unspliced CDKN1A. *P* value(si#21, UT) = 0.00001; *P* value(si#21, 1 h) = 0.000003; *P* value(si#21, 2 h) = 0.0001; *P* value(si#21, 12 h) = 0.009. *P* value(si#22, UT) = 0.05. Data are presented as mean ± s.d. (*n* = 3 biologically independent experiments). All the statistical tests are two-tailed, tow-sided *t-test*. *p < 0.05, **p < 0.01, ***p < 0.001, ****p < 0.0001. Source data are provided as a Source Data file.

Prompted by the aforementioned results, we sought to investigate the regulation of p21 levels by RBM42 during DNA damage. Western blot analysis revealed that p21 protein levels are noticeably lower in RBM42-deficient HCT116 compared to control cells following treatment with various concentrations of VP16 (Fig. 2a; Supplementary Fig. 2a). Similar effect on p21 levels was also observed following RBM42 depletion in U2OS cells, suggesting that the reduction in p21 levels following RBM42 depletion is not cell-type specific (Supplementary Fig. 2b). Consequently, RBM42 depletion leads to a decrease in cell viability, which was accompanied by defective DNA damage-induced G1/S arrest, and elevated levels of apoptosis (Fig. 2b-d; Supplementary Fig. 2c). Moreover, similar to p21 deficiency, RBM42 depletion hypersensitizes cells to VP16-induced DNA damage and to WEE1 inhibition, which is known to override G2/M checkpoint (Fig. 2e-f). Collectively, our data show that RBM42 deficiency recapitulates some of the phenotype observed following p21 downregulation^31–34^.

Interestingly, RBM42-deficient cells show prominent increase in p53 protein levels compared to RBM42-proficient cells (Fig. 2a; Supplementary Figs. 2a-b). This finding indicates that the lack of p21 induction in VP16-treated RBM42-deficient cells is not due to decrease in p53 protein levels. Next, we sought to test a possibility that RBM42 regulates p53 binding to CDKN1A promoter region, which is known to be essential for CDKN1A transcription in response to DNA damage^9,35,36^. We performed p53 chromatin immunoprecipitation followed by quantitative PCR (ChIP-qPCR) in RBM42-proficient and -deficient cells, before and after DNA damage. As previously reported^16,37^, p53 protein levels at CDKN1A promoter were increased following DNA damage (Fig. 2g). Interestingly, our results show that p53 binding to CDKN1A promoter is even further enhanced following RBM42 deficiency (Fig. 2g). Moreover, ChIP-qPCR analysis showed that RBM42 is not recruited to CDKN1A promoter region, arguing against a possibility of a direct role of RBM42 in regulating CDKN1A transcription (Supplementary Fig. 2d). Concordantly, quantitative reverse transcriptase PCR (qRT-PCR) analysis showed increase in the levels of CDKN1A unspliced transcript following RBM42 depletion, irrespective of DNA damage induction, suggesting that RBM42 is not required for CDKN1A transcription (Fig. 2h; Supplementary Figs. 2e-h). Moreover, we inferred that the increase in CDKN1A transcription upon RBM42 depletion in untreated cells is likely due to the activation of DDR, as evident by elevated levels of γH2AX and p53 pathway-related genes (Fig. 1a; 2a; Supplementary Fig. 1d). Altogether, our data suggest that the defective induction of p21 protein levels in VP16-treated RBM42-deficient cells is independent of p53 transcription activity, and it is likely due to abnormal posttranscriptional processing of CDKN1A transcript.

### RBM42 regulates CDKN1A splicing during DNA damage

**Fig. 3:**
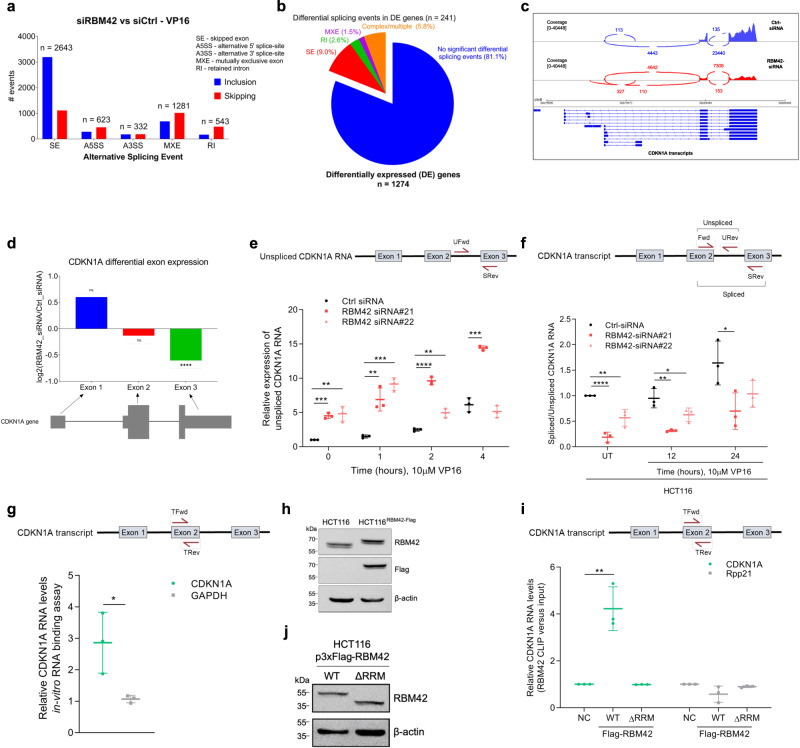
RBM42 regulates CDKN1A splicing during DNA damage. **a** Summary of significant alternative splicing events (leading to skipping (red) or inclusion (blue) observed upon RBM42 depletion in VP16-treated cells as detected by rMATS^84^. Significantly altered splicing events were classified as having a minimum inclusion level difference of 0.1, *p* value < 0.01, and FDR < 0.01. SE: skipped exon, MXE: mutually exclusive exons, A5SS: alternative 5’ splice site, A3SS: alternative 3’ splice site, IR: intron retention. **b** Pie chart showing the distribution of significant alternative splicing events among the differentially expressed genes (identified in Fig. 1b) upon RBM42 depletion in VP16-treated cells. **c** Representative sashimi plot showing RNA-seq read coverage across CDKN1A gene. Arcs correspond to reads spanning exon-exon junctions and number of reads corresponding to junctions is shown. CDKN1A isoforms are shown below. **d** Differential expression of CDKN1A exons upon RBM42 depletion as quantified by DEXSeq^85^. **p < 0.001, ***p < 0.0001, ns: not significant. *P* value (Exon3)<0.0001. **e** RBM42 depletion increases the levels of CDKN1A unspliced transcript containing exon 3 during damage. (Top) PCR primer locations. *P* value*:* (si#21, UT) = 0.0001; (si#21, 1 h)=0.005; (si#21, 2 h)=0.00003; (si#21, 4 h)=0.0002; (si#22, UT) = 0.004; (si#22, 1 h)=0.0002; (si#22, 2 h) = 0.003 **f** RT-qPCR shows that RBM42 knockdown disrupts CDKN1A splicing. Graph shows the ratio between the relative expression of spliced and unspliced CDKN1A transcripts. (Top) Schematic showing qPCR primers used to detect unspliced or spliced CDKN1A transcript. P-*value:* (si#21, UT) = 0.0001; (si#21, 12 h)=0.004; (si#21, 24 h)=0.05; (si#22, UT) = 0.009; (si#22, 12 h)=0.05. **g** In vitro RNA binding shows that GST-RBM42 binds CDKN1A RNA. Precipitated RNA was quantified by qRT-PCR. (Top) qPCR primers used to detect CDKN1A transcript. Values were normalized to GST-Only. *P* value = 0.03. **h** Western blot validating the biallelic knock-in of flag at the endogenous RBM42 coding sequence. **i** CLIP-qPCR showing binding of RBM42 to CDKN1A RNA via its RRM domain. HCT116 cells expressing either RBM42 wild type or mutant lacking RRM domain (ΔRRM) were subjected to CLIP-qPCR. P-*value*(WT) = 0.004. **j** Western blot shows comparable levels of RBM42 wild type and Flag-ΔRRM mutant. Data are presented as mean ± s.d. (*n* = 3 biologically independent experiments). Two-tailed *t-test*. *p < 0.05, **p < 0.01, ***p < 0.001, ****p < 0.0001. Source data are provided as a Source Data file.

Since human RBM42 is a component of the spliceosomal pre-B complex^26^, we hypothesized that RBM42 regulates RNA splicing, and specifically CDKN1A splicing, during DNA damage. Indeed, genome-wide splicing analysis revealed thousands of differential alternative splicing events affecting ~4500 genes upon RBM42 depletion before and after DNA damage (FDR < 0.05), with exon inclusion being the primary alternative splicing event (Fig. 3a; Supplementary Fig. 3b; Supplementary Data 2-3). To confirm the authenticity of the RNA splicing analysis, we depleted RBM42 using two different siRNA sequences and measured the splicing of 5 target genes identified by RNA-seq. qRT-PCR analysis showed that the splicing efficiency of all tested genes was reduced following RBM42 depletion, confirming the regulatory effect of RBM42 on splicing (Supplementary Figs. 3d-h). Interestingly, we observed that ~20% of the differentially expressed genes exhibit changes in splicing patterns, suggesting that RBM42 regulates gene expression in a splicing-dependent and -independent manner (Fig. 3b; Supplementary Fig. 3c). Differential KEGG pathway analysis shows that RBM42 splicing targets are enriched in multiple pathways including RNA splicing, translation, cell cycle regulation and p53 signaling pathways (Supplementary Fig. 3a). Since exon inclusion/skipping constitutes the majority of alternative splicing events observed upon RBM42 depletion, we estimated the differential exon expression in RBM42-depleted cells compared with control cells (Supplementary Data 4). Interestingly, we found that RBM42 depletion alters the expression levels of CDKN1A exons, leading to a significant decrease exclusively in the last exon (exon 3), as well as decrease usage of exon 2-exon 3 spliced junction (Fig. 3c-d). These results raise a possibility that RBM42 is involved in CDKN1A transcription elongation or RNA splicing of exon 3. To test this, we measured the levels of unspliced CDKN1A using primers that flank intron 2 and exon 3 in RBM42-proficient and deficient cells. Results showed elevated levels of the unspliced CDKN1A transcript containing exon 3 following RBM42 depletion, suggesting that RBM42 is not involved in regulating CDKN1A exon 3 transcription elongation (Fig. 3e). Subsequently, we employed qRT-PCR to determine the splicing efficiency of CDKN1A, using exon-exon and intron-exon junction-specific primers, flanking exon 2 and exon 3. Results showed that RBM42 depletion disrupts CDKN1A splicing during DNA damage inflicted by VP16 and ionizing radiation (IR) in HCT116 and U2OS cells (Fig. 3f; Supplementary Figs. 4a-e). To substantiate RBM42 role in regulating CDKN1A splicing, we tested the effect of RBM42 depletion on the splicing of CDKN1A using minigene reporter including genomic sequence corresponding to exon 2-exon 3 region. Results show that RBM42 depletion disrupts the splicing CDKN1A minigene, consistent with RBM42 effect on the endogenous CDKN1A splicing (Supplementary Fig. 4g). To further confirm that the decrease in the spliced CDKN1A upon RBM42 depletion is due to defective splicing and not the introduction of alternative polyadenylation site, we performed 3’ Rapid Amplification of cDNA Ends (RACE) to map potential polyadenylation variants of endogenous CDKN1A and CDKN1A minigene. We observed that RBM42 depletion does not lead to the formation of new polyadenylated CDKN1A variants (Supplementary Fig. 4h-i). Moreover, we show that RBM42 depletion has no detectable effect on the stability of CDKN1A mRNA (Supplementary Fig. 4f). Collectively, we concluded that RBM42 depletion leads to defective splicing of CDKN1A, which contributes, at least partially, to the low protein levels of p21 observed after DNA damage. Next, we sought to determine the effect of RBM42 depletion on the splicing of other p53 target genes, such as GADD45, MDM2 and PUMA. Contrary to CDKN1A, the splicing efficiency of these genes remains intact in RBM42-deficient cells during DNA damage (Supplementary Figs. 5a-c). These findings are in line with RBM42 transcriptome showing that the expression of GADD45, MDM2 and PUMA is not compromised following RBM42 depletion (Fig. 1d; Supplementary Data 1). Collectively, our data identified human RBM42 as a splicing regulator, and CDKN1A as a splicing target of RBM42 during DNA damage.

Next, we showed that RBM42 binds CDKN1A RNA during DNA damage using two complementary approaches. First, we purified GST-RBM42 fusion protein and showed that it binds CDKN1A RNA in vitro (Fig. 3g). Second, we used crosslinking and immunoprecipitation **(**CLIP) to test RBM42 interaction with CDKN1A transcript in cells. Toward this end, we used CRISPR-Cas9 technology to knock-in 3xflag tag at the 3’-end of the endogenous RBM42 gene to establish HCT116 cell line expressing RBM42-flag fusion (HCT116^RBM42-Flag^). Interestingly, western blot showed that the two splicing isoforms of RBM42 observed in HCT116 control cells were also observed in HCT116^RBM42-Flag^, suggesting that the addition of flag to RBM42 C-terminal has no detectable effect on RBM42 splicing (Fig. 3h). Next, VP16-treated HCT116 and HCT116^RBM42-Flag^ cells were subjected to CLIP-qPCR using flag antibody. Results showed that RBM42 directly binds CDKN1A RNA (Fig. 3i). Additionally, CLIP assay showed that RBM42 deletion mutant, that lacks the RRM domain, lost its ability to bind CDKN1A RNA (Fig. 3i-j). Altogether, we concluded that RBM42 directly binds CDKN1A RNA through its RRM domain, and thus supporting the notion that CDKN1A is a bona fide splicing target of RBM42.

### RBM42 interactome substantiates its function as a splicing regulator

**Fig. 4:**
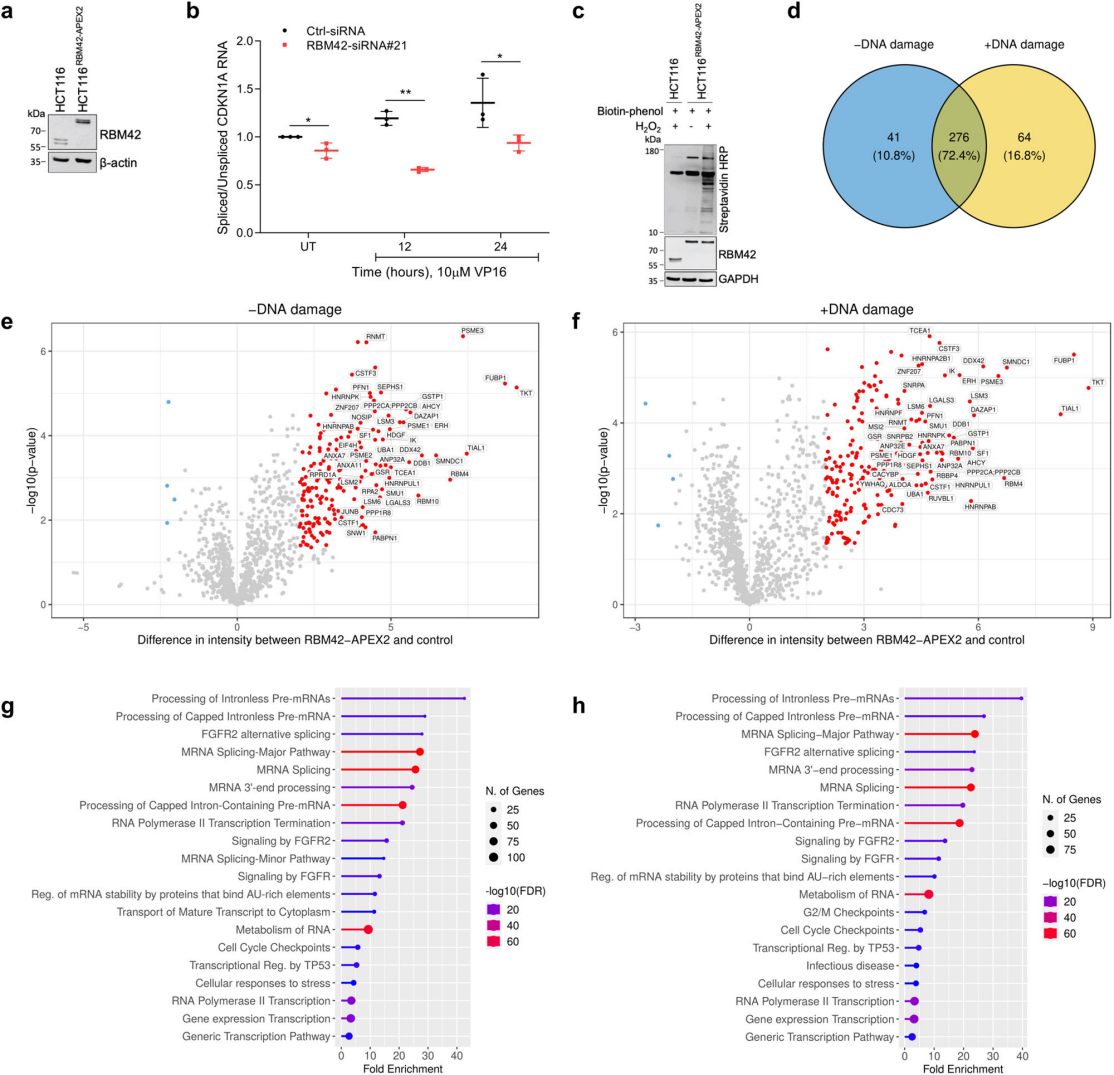
RBM42 interactome substantiates its function as a splicing regulator. **a** Western blot analysis validating the establishment of biallelic knock-in of APEX2 at the C-terminus of the endogenous RBM42 coding sequence in HCT116 cells. Protein samples were prepared from control and HCT116^RBM42-APEX2^ cells and immunoblotted using the indicated antibodies. **b** qRT-PCR analysis, as in Fig. 3f, showing the effect of RBM42-APEX2 depletion on CDKN1A splicing before and after DNA damage in HCT116^RBM42-APEX2^ cells. *P* *value:* (UT) = 0.03; (12 h) = 0.0003; (24 h) = 0.05. Data are presented as mean ± s.d. (*n* = 3 biologically independent experiments). All the statistical tests are two-tailed *t-test*. *p < 0.05, **p < 0.01, ***p < 0.001. **c** Western blot analysis confirming APEX2 functionality in HCT116^RBM42-APEX2^ cells. Control and HCT116^RBM42-APEX2^ cells were incubated with 0.5 mM Biotin-phenol for 2 h and activated using 1 mM H2O2 for 2 min and subjected to western blot using streptavidin–HRP antibody to detect biotinylated proteins. **d** Venn diagram plot showing the intersection of the proteins significantly enriched in proximity to RBM42 before and after DNA damage induction by treatment with 10 µM VP16 for 24 h. Proteins with the difference in mean intensity > 1 and FDR < 0.05 between HCT116^RBM42-APEX2^ and control cells were classified as significantly enriched. **e**, **f** Volcano plots showing the proteins enriched in proximity to RBM42 before (**e**) and after (**f**) DNA damage induction. Highly enriched proteins with intensity difference > 2 and *p* value < 0.05 are indicated in red. Statistical test is students *t-test.* **g**, **h** Pathway enrichment analysis (Reactome database) of proteins significantly enriched in proximity to RBM42 before (**g**) and after (**h**) DNA damage induction. Source data are provided as a Source Data file.

**Fig. 5:**
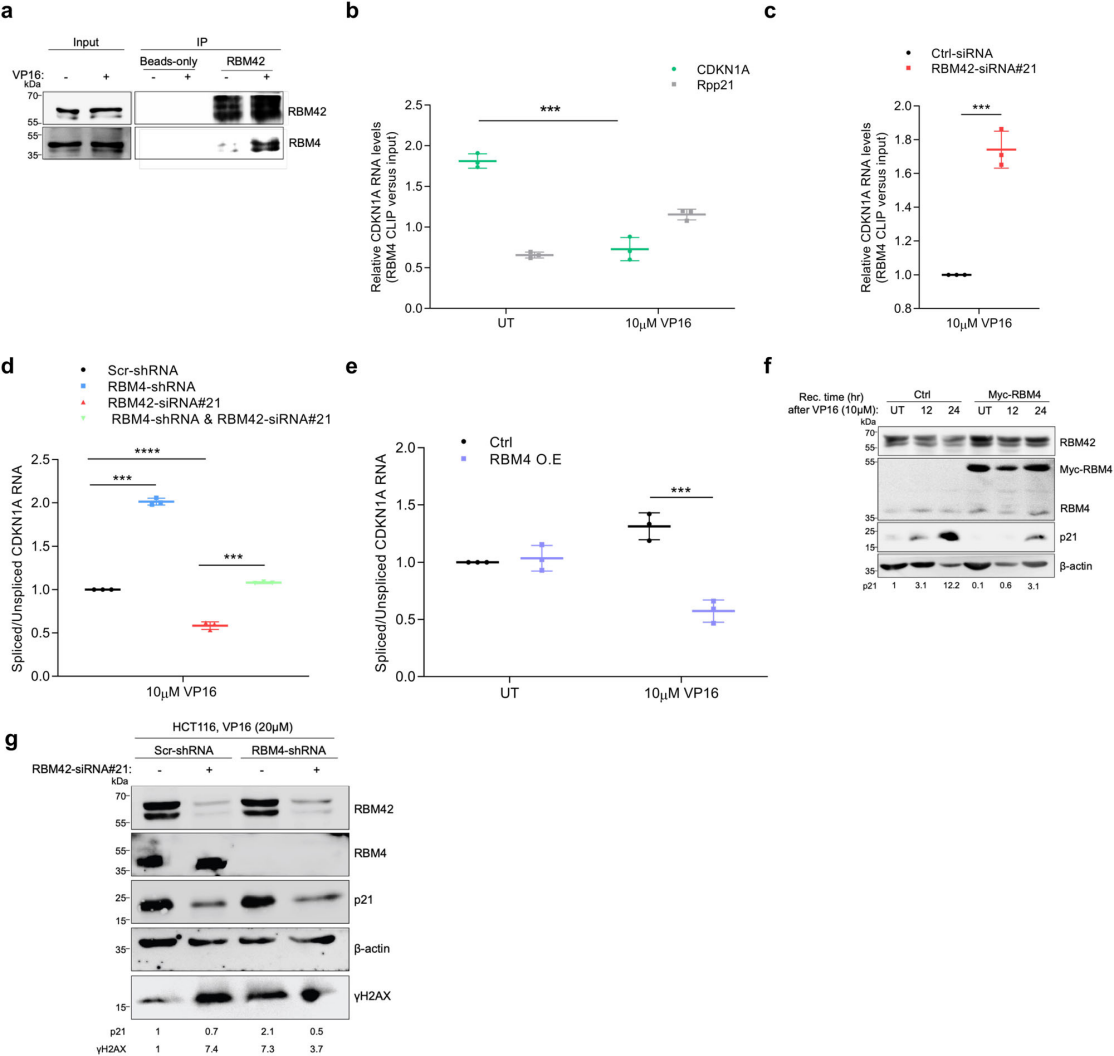
RBM42 regulates CDKN1A splicing during DNA damage via counteracting RBM4 activity. **a** Immunoprecipitation of endogenous RBM42 in HCT116 cells shows RBM42 interaction with RBM4. Whole-cell lysates were prepared from HCT116 cells treated with 10μM VP16 for 18 h or left untreated (UT). Lysates were subjected to immunoprecipitation using RBM42 antibody or beads-only and subjected to immunoblot analysis with the indicated antibodies. **b**, **c** CLIP-qPCR shows binding of RBM4 to CDKN1A RNA in HCT116 before and after VP16 treatment (**b**), and in RBM42-deficient cells treated with VP16 *P* value = 0.0004 (**c**). CLIP-qPCR was performed as in Fig. 3i, except of using control and HCT116 cells expressing myc-RBM4 that were treated with 10 µM VP16 for 18 h or left untreated. *P* value = 0.0003. **d** Co-depletion of RBM4 and RBM42 restores CDKN1A splicing integrity. RT-qPCR, as in Fig. 3f, shows the splicing efficiency of CDKN1A in control and HCT116 cells depleted either of RBM42, RBM4, or co-depleted of RBM42 and RBM4. P value: (RBM4-sh)=0.0003; (si#21) = 0.00008; (si#21 and RBM4-sh)=0.0001 **e** Overexpression of myc-RBM4 counteracts CDKN1A splicing during DNA damage. RT-qPCR, as in Fig. 3f, shows the splicing efficiency of CDKN1A. *P* value (VP16) = 0.001. **f** Western blot analysis shows that myc-RBM4 overexpression impairs the DNA damage-induced increase of p21 protein levels. Protein extracts of untreated and VP16-treated HCT116 cells expressing myc-only or myc-RBM4 were prepared as in Fig. 1a, and immunoblotted with the indicated antibodies. Band intensities of p21 were normalized to the intensities of their respective β-actin bands and are shown at the bottom of the blot. Protein molecular weight sizes are indicated at the left side of the western blots. **g** Western blot shows p21 expression following VP16 treatment in HCT116 cells expressing either scramble or RBM4 shRNA and transfected with control (Ctrl) or RBM42 siRNA. Band intensities were measured as in (5 f). Data are presented as mean ± s.d. (*n* = 3). All the statistical tests are two-tailed *t-test*. *p < 0.05, **p < 0.01, ***p < 0.001. Source data are provided as a Source Data file.

To gain deeper insights into how RBM42 regulates RNA splicing, we sought to map RBM42 interactome using ascorbate peroxidase (APEX2)-based proximity labelling combined with mass spectrometry^38^. We utilized CRISPR-Cas9 methodology for generating biallelic knock-in of ascorbate peroxidase (APEX2) at the C-terminus of the endogenous RBM42 coding sequence (CDS) and established an HCT116 cell line expressing RBM42-APEX2 fusion, hereafter named HCT116^RBM42-APEX2^ (Supplementary Figs. 6a-b). As in control HCT116 cells, western blot shows two bands of RBM42-APEX2 fusion corresponding to its two splicing variants, suggesting that the addition of APEX2 to RBM42 C-terminal has no deleterious effect on RBM42 splicing (Fig. 4a). Moreover, the splicing activity of RBM42 is not compromised following the fusion of APEX2 to its C-terminus, as determined by measurement of CDKN1A splicing efficiency (Fig. 4b). We concluded therefore that HCT116^RBM42-APEX2^ cells express functional RBM42. Next, we sought to validate that HCT116^RBM42-APEX2^ cells express functional APEX2 that can biotinylate nearby proteins. To determine APEX2 functionality, control and HCT116^RBM42-APEX2^ cells were grown in the absence and presence of biotin phenol, followed by H_2_O_2_ treatment to activate APEX2, and western blot using streptavidin-HRP that recognizes biotinylated proteins. Results show that H_2_O_2_-treated HCT116^RBM42-APEX2^ cells exhibits prominent biotinylated bands when compared to either H_2_O_2_-untreated HCT116^RBM42-APEX2^ and H_2_O_2_-treated control HCT116 cells (Fig. 4c). Altogether, these results confirmed the functionality of both RBM42 and APEX2 and pave the way for mapping RBM42 interactome during DNA damage. To map RBM42 interactome, control and HCT116^RBM42-APEX2^ cells were treated with DMSO or VP16 and incubated in media containing biotin phenol, followed by H_2_O_2_ treatment, to activate APEX2 peroxidase activity. Next, cell lysates were enriched for biotinylated proteins and subjected to mass spectrometry (MS) analysis (Supplementary Fig. 6c). We identified 340 and 317 proteins significantly enriched in proximity to RBM42 before and after DNA damage, respectively (Fig. 4d-f; Supplementary Data 5). Next, we used immunoprecipitation as an orthogonal approach to test RBM42 interaction with selected proximal proteins. Results showed that RBM42 interacts with RBM4, CUGBP1, LSM4, C8orff33 and hnRNP K, confirming the authenticity of RBM42 interactome (Fig. 5a; Supplementary Fig. 6d and see below Fig 7a). Pathway enrichment analysis revealed that the majority of proteins that appeared in proximity to RBM42 are implicated in RNA processing including splicing (Fig. 4g-h). Interestingly, we also observed significant enrichment of proteins involved in cell cycle checkpoints and regulation, further highlighting the role of RBM42 in DDR (Fig. 4g-h). Together, RBM42 interactome analysis substantiates its function as a splicing regulator and implicates it in additional cellular pathways related to mRNA metabolism and DDR.

### RBM42 regulates CDKN1A splicing during DNA damage via counteracting RBM4 activity

Despite the potential value of all RBM42-interacting partners, we focused on the splicing inhibitor RBM4^39^, since it appears among the highly enriched RBM42-interacting proteins (Fig. 4e-f; Fig. 5a) and was shown to directly bind CDKN1A RNA by photoactivatable ribonucleoside-enhanced crosslinking and immunoprecipitation (PAR-CLIP)^40^. To study a potential crosstalk between RBM42 and RBM4 in regulating CDKN1A splicing, we first performed RBM4 CLIP followed by qRT-PCR and showed that RBM4 directly interacts with CDKN1A transcript, as previously described^40^ (Fig. 5b). Next, we found that contrary to RBM42, RBM4 binding to CDKN1A RNA is reduced following DNA damage (Fig. 5b; Supplementary Fig. 7a). Remarkably, RBM42 depletion suppresses the DNA damage-induced reduction of RBM4 binding to CDKN1A RNA, suggesting that RBM42 counteracts RBM4 binding to CDKN1A transcript after DNA damage (Fig. 5c). Afterward, we sought to determine the effect of RBM4 on CDKN1A splicing. Results showed that RBM4 knockdown increases in CDKN1A splicing efficiency (Fig. 5d; Supplementary Figs. 7b-d). Consistently, RBM4 overexpression leads to a reduction in CDKN1A splicing efficiency and p21 protein levels, which recapitulates RBM42 deficiency (Fig. 5e-f). Altogether, our findings confirm the opposing roles of RBM42 and RBM4 in regulating CDKN1A splicing during DNA damage.

Since RBM4 and RBM42 proteins have opposite outcome on CDKN1A splicing, and since RBM42 attenuates RBM4 binding to CDKN1A RNA following DNA damage, we sought to determine the impact of RBM42 and RBM4 co-depletion on CDKN1A splicing. Our results showed that simultaneous depletion of RBM42 and RBM4 restores CDKN1A splicing to its normal level similar to control cells (Fig. 5d; Supplementary Fig. 7b). We concluded therefore that RBM42 regulates CDKN1A splicing, at least partly, by counteracting the splicing inhibitory activity of RBM4. As a control, we tested the effect of either depletion of RBM4, RBM42, or both RBM4 and RBM42 on Rpp21 splicing. Results show that neither depletion of RBM42, RBM4, nor RBM42 and RBM4 affect Rpp21 splicing (Supplementary Fig. 7e). These results further highlight the specific antagonistic crosstalk between RBM4 and RBM42 in regulating CDKN1A splicing. Collectively, our findings provide firm evidence that RBM42 regulates CDKN1A splicing during DNA damage by counteracting RBM4 binding to CDKN1A RNA and thereby neutralizing its inhibitory effect on CDKN1A splicing.

### RBM42 underpins CDKN1A translation during DNA damage

**Fig. 6:**
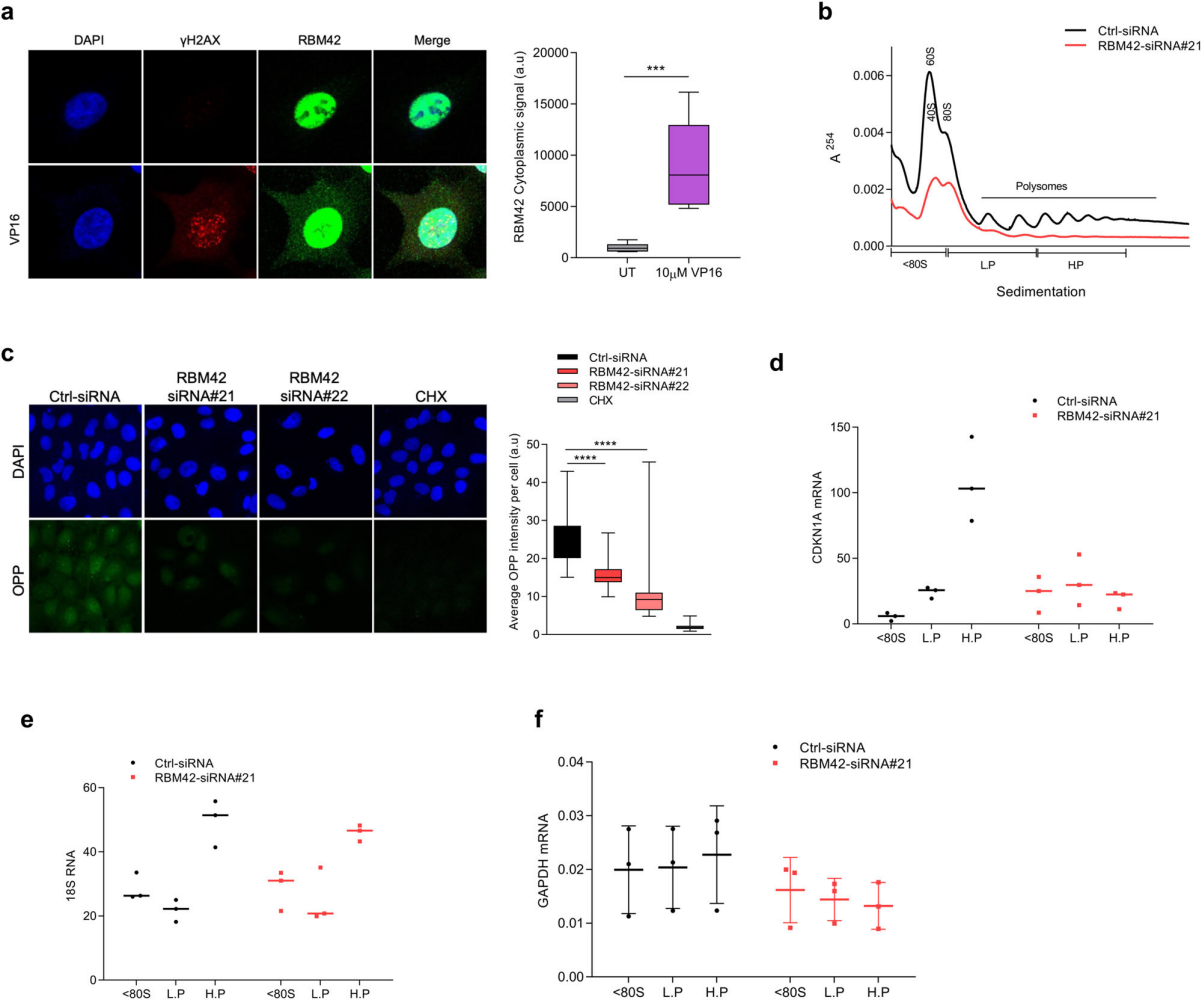
RBM42 underpins CDKN1A translation during DNA damage. **a** Representative immunofluorescence image showing RBM42 subcellular localization (Left). Untreated and VP16-treated HCT116 cells were stained with RBM42 (green) and γH2AX antibody (red), and DNA is stained with DAPI (blue). Graph shows quantification of RBM42 cytoplasmic signal using ImageJ (Right). *P* value = 0.001. **b** Graph shows absorbance profile of ribosomes at 254 nm. Absorbance peaks representing polysomes are indicated. Lysates were prepared from U2OS cells transfected with control (Ctrl) or RBM42 siRNA and treated with 5 μM VP16 for 18 h. lysates were fractionated over a 10-50% sucrose density gradient. **c** OPP assay shows that RBM42 depletion inhibits protein translation. U2OS cells transfected with control (Ctrl) or RBM42 siRNA and treated with 5 μM VP16 for 18 h were subjected to OPP assay. U2OS cells treated with cycloheximide (CHX) are used as a negative control. **d**–**f** RT-qPCR analysis to determine the RNA abundance of CDKN1A (d), 18S (**e**) and GAPDH (**f**) in the polysomal fractions. Graphs show the ratio between the relative expression of the RNA in each of the indicated fractions relative RNA expression in all fractions together (overall expression). Data are presented as mean ± s.d. (*n* = 3 biologically independent experiments). All the statistical tests are two-tailed *t-test*. *p < 0.05, **p < 0.01, ***p < 0.001. L.P and H.P correspond to light and heavy polysomes, respectively. Source data are provided as a Source Data file.

Since RBM4 depletion amended the defect of CDKN1A splicing in VP16-treated RBM42-deficient cells (Fig. 5d; Supplementary Fig. 6a), we sought to determine whether p21 protein levels are also restored to normal levels as in control cells. Unexpectedly, we observed reduced p21 protein levels in VP16-treated HCT116 cells co-depleted of RBM42 and RBM4 compared to control cells (Fig. 5g). These results raise a possibility that beside splicing, RBM42 regulates CDKN1A translation. In support of this, RBM42 interactome data revealed several proteins involved in translation, such as CUG binding protein 1 (CUGBP1) in proximity to RBM42 (Supplementary Data 6). Moreover, immunofluorescence (IF) analysis showed that while RBM42 is enriched in the nucleus, its cytoplasmic localization increases following DNA damage, and therefore it is conceivable that it might be involved in translation regulation (Fig. 6a). To test this, we applied sucrose gradient polysome fractionation in RBM42-proficient and -deficient cells treated with VP16. Results showed significant reduction in ribosome abundance following RBM42 depletion, as evident by the reduction in 80 S and 40 S/60 s peaks and the flattened polysome peak amplitude compared to control cells, suggesting that RBM42 has a role in translation regulation during DNA damage (Fig. 6b). Results showed significant reduction in ribosome abundance following RBM42 depletion, as evident by the reduction in 80 S and 40 S/60 s peaks and the flattened polysome peak amplitude compared to control cells, suggesting that RBM42 has a role in translation regulation during DNA damage (Fig. 6b). To further substantiate the effect of RBM42 on global translation, we measured the levels of nascent protein synthesis using O-propargyl puromycin (OPP) assay after VP16 treatment. Results show that RBM42 depletion leads to a prominent decrease in protein synthesis, consistent with the polysomal profile (Fig. 6c). Next, we measured the relative abundance of CDKN1A mRNA in 15 ribosomal fractions collected from the sucrose gradient. We found that CDKN1A mRNA shifts to lighter polysomal fractions following RBM42 depletion, indicating that CDKN1A mRNA is less translated (Fig. 6d). On the other hand, RBM42 depletion doesn’t affect 18S and GAPDH abundance across the polysomal fractions, indicating that their translation is independent of RBM42 (Fig. 6e-f). Altogether, polysome profiling qRT-PCR analysis suggests that RBM42 is required for efficient CDKN1A translation during DNA damage.

### RBM42-CUGBP1 axis regulates CDKN1A translation during DNA damage

**Fig. 7:**
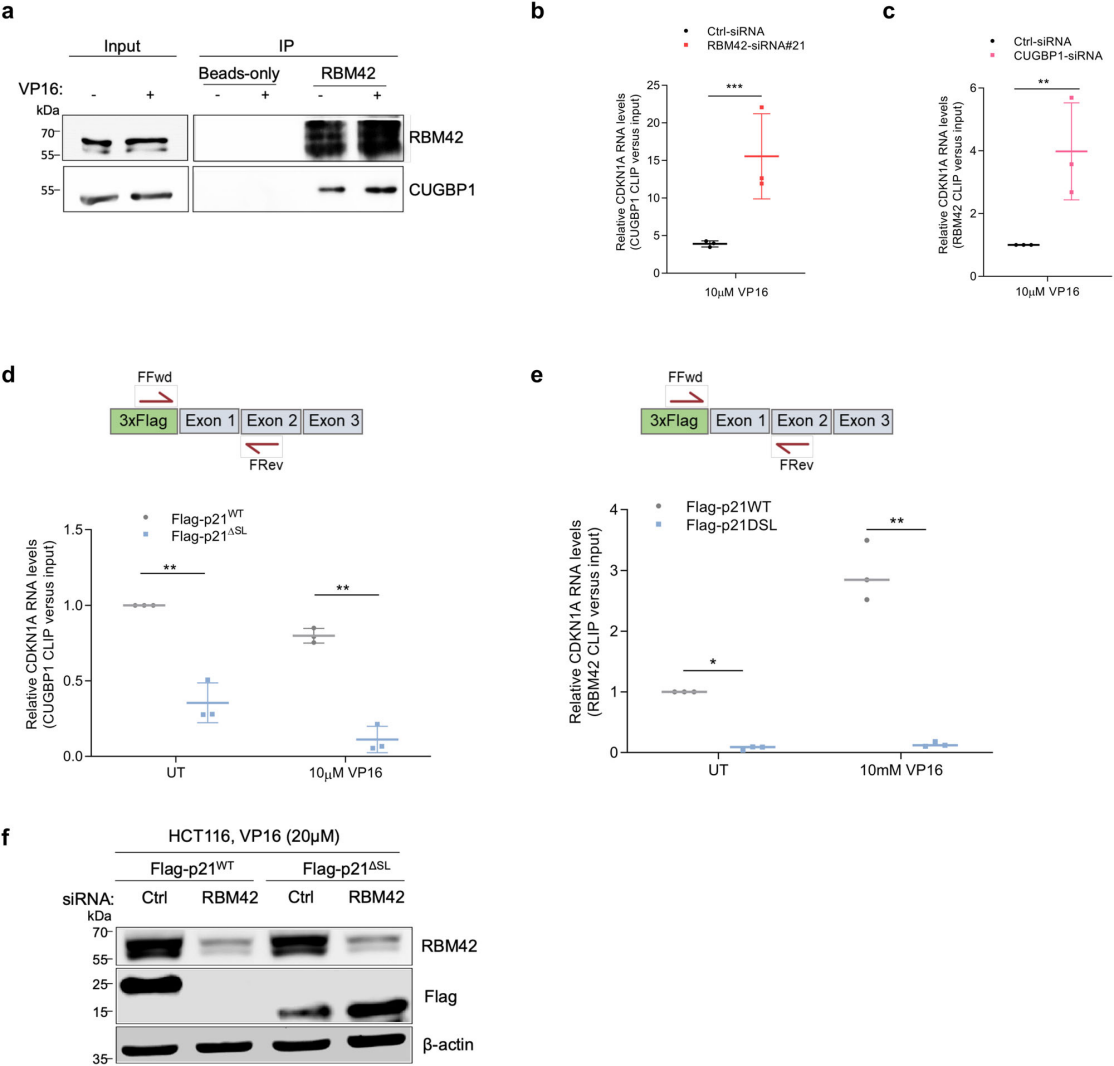
RBM42-CUGBP1 axis regulates CDKN1A translation during DNA damage. **a** Shows RBM42-CUGBP1 interaction by immunoprecipitation of endogenous RBM42 as in Fig. 5a. **b** CLIP-qPCR, as in Fig. 3i, shows the binding of CUGBP1 to CDKN1A RNA following RBM42 depletion. Control and HCT116 cells expressing myc-CUGBP1 were transfected with control (Ctrl) or RBM42 siRNA and treated with 10 μM VP16 for 18 h. **c** CLIP-qPCR, as in (**b**), shows the binding of RBM42 to CDKN1A RNA following CUGBP1 depletion. **d**, **e** CLIP-qPCR shows the binding of CUGBP1 and RBM42 to Flag-p21^WT^ and Flag-p21^ΔSL^ transcripts following VP16 treatment. Control and HCT116 cells expressing myc-CUGBP1 were transfected with vectors expressing Flag-p21^WT^ or 3xFlag-p21^ΔSL^. Next, cells were treated with 10 μM VP16 for 18 h or left untreated (UT) and subjected to CLIP-qPCR. Data are presented as mean ± s.d. (n = 3 biologically independent experiments). *p < 0.05, **p < 0.01, ***p < 0.001. **f** Western blot analysis shows Flag-p21^WT^ and Flag-p21^ΔSL^ protein levels in control and RBM42-deficient cells. Source data are provided as a Source Data file.

To shed molecular insights into how RBM42 regulates CDKN1A translation, we took advantage of RBM42 interactome, which revealed several translation factors in proximity to RBM42 (Supplementary Data 5). We focused on CUGBP1^41^, since it was shown to promote CDKN1A translation in undamaged cells, via binding to a stem and loop (SL) sequence located at the 5’ region of CDKN1A coding sequence (CDS)^17,42^. First, we confirmed the interaction between RBM42 and CUGBP1 (Fig. 7a). Next, we examined a potential crosstalk between RBM42 and CUGBP1 on binding CDKN1A RNA. CLIP showed that upon DNA damage RBM42 depletion enhances CUGBP1 binding to CDKN1A RNA (Fig. 7b). Also, CUGBP1 depletion enhances RBM42 binding to CDKN1A RNA (Fig. 7c). We assumed therefore that the binding of RBM42 and CUGBP1 to CDKN1A transcript is regulated by the same SL RNA sequence at the 5’ region of CDKN1A CDS. To test this assumption, we performed CLIP for RBM42 and CUGBP1 in HCT116 cells expressing flag fused to either wild-type CDKN1A CDS (Flag-p21^WT^) or to in-frame deletion mutant of CDKN1A CDS lacking the SL sequence (Flag-p21^ΔSL^). Our results show that both RBM42 and CUGBP1 binds Flag-p21^WT^ but not Flag-p21^ΔSL^ RNA (Fig. 7d-e; Supplementary Fig. 8a). Therefore, we concluded that the SL region of CDKN1A is critical for RBM42 and CUGBP1 binding. Interestingly, we noticed that while DNA damage suppresses CUGBP1 binding to CDKN1A RNA, it enhances RBM42 binding, suggesting that CUGBP1 and RBM42 bind CDKN1A RNA under different cellular conditions (Fig. 7d-e). Next, we sought to decipher the functional relevance of RBM42 binding to the SL region of CDKN1A RNA. Since CUGBP1 binds the SL region of CDKN1A and promotes its translation before DNA damage, we suspected that RBM42 may have a similar function after DNA damage. Toward this end, control and RBM42-deficient cells were transfected with constructs expressing either Flag-p21^WT^ or Flag-p21^ΔSL^ and then treated with VP16. Western blot showed that the protein levels of Flag-p21^WT^, but not Flag-p21^ΔSL^, are significantly reduced upon RBM42 knockdown (Fig. 7f; Supplementary Fig. 8b). Collectively, these results confirm our prediction that the SL region is required for regulating CDKN1A translation by RBM42 following DNA damage.

### Genome-wide mapping of RBM42 RNA binding sites during DNA damage

**Fig. 8:**
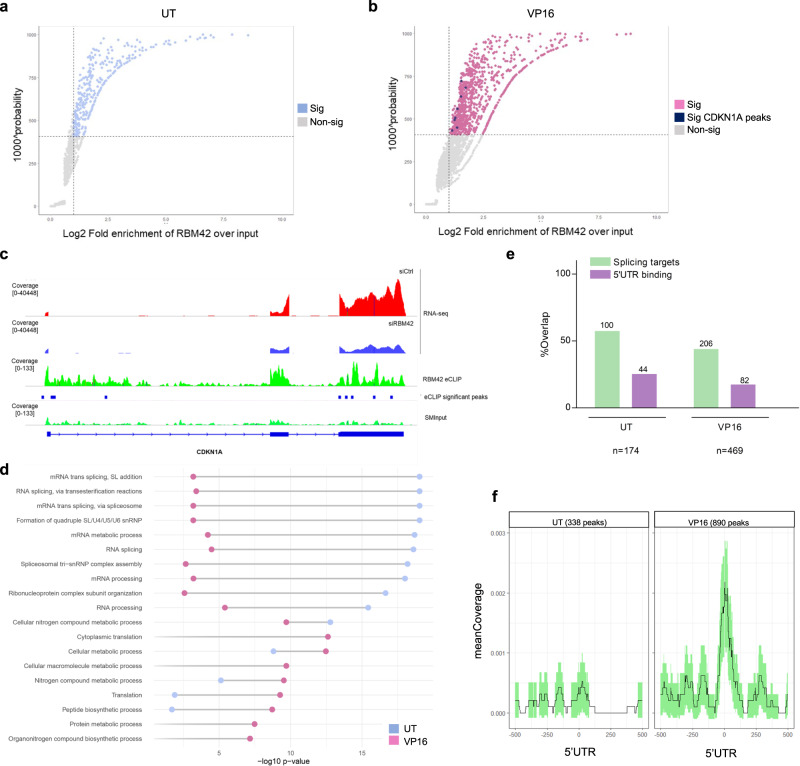
Transcriptome-wide mapping of RBM42-RNA interactions during DNA damage. **a**, **b** Results from RBM42 eCLIP-seq using three biological replicates before (**a**) and after (**b**) DNA damage. A size-matched input (SMInput), and a non-CL sample. Light blue (before damage) and pink (after damage) dots represent enriched peaks defined as those with more than 2-fold enrichment in eCLIP over control (SMInput + non-CL) and probability above 0.87. p21 peaks are marked in blue (**b**). Only peaks with fold enrichment > 2 are shown. **c** Representative RNA-seq and eCLIP-seq read coverage tracks of CDKN1A gene. Significant eCLIP peaks (Probability >0.87) are indicated by blue boxes. **d** Pathway enrichment analysis of genes that were found by eCLIP to interact with RBM42 before (blue dots) and after VP16 treatment (pink dots). **e** Shows percent overlap between RBM42-bound transcripts and RBM42 splicing targets before and after VP16 treatment. Green bars correspond to alternatively spliced transcripts directly bound by RBM42. Purple bars correspond to alternatively-spliced transcripts that exhibit RBM42 binding at the 5’UTR. The number of transcripts associated with each group are indicated on top of the bars. The number of all RBM42-bound transcripts are indicated at the bottom of the graph. **f** Shows the increase in RBM42 binding at the 5’UTR following DNA damage induction by VP16. Source data are provided as a Source Data file.

To shed further molecular insights into the emerging dual role of RBM42 in regulating splicing and translation machineries, we sought to map RNA sequences that are directly bound to RBM42 before and after DNA damage. To achieve this, we used an enhanced crosslinking and immunoprecipitation followed by high-throughput RNA sequencing (eCLIP)^43,44^. The eCLIP results revealed a total of 338 and 890 significant peaks (Probability> 0.87, Fold change > 2) before and after DNA damage, respectively (Fig. 8a-b; Supplementary Data 6). Importantly, the eCLIP data confirmed the direct binding of RBM42 to CDKN1A transcript after DNA damage (Fig. 8b) Indeed, RBM42 binds CDKN1A transcript from the 5’UTR throughout the end of the second exon. Additionally, we found an enrichment of four distinct binding sites at the 3’UTR corresponding to exon 3 which was downregulated upon RBM42 depletion (Fig. 8c). This binding pattern further supports a role of RBM42 in regulating splicing and translation of CDKN1A. eCLIP peaks analyses led to several observations. First, annotation of RBM42 eCLIP peaks showed that RBM42 extensively binds various spliceosome components before and after DNA damage. For example, RBM42 binds U6 snRNA, a core component of the catalytic spliceosome that is critical for RNA splicing^45^ (Fig. 8d). These observations provide molecular insights into the splicing regulatory role of RBM42, and are in line with previous works that have identified RBM42 by mass-spectrometry as an integral component of the tri-snRNP complex^26^. Second, 50% of the transcripts bound by RBM42 exhibit alterations in splicing following RBM42 depletion, suggesting that they are direct splicing target genes of RBM42 (Fig. 8e; Supplementary Data 6). Third, upon DNA damage, RBM42 binding is markedly enriched at the 5′UTR of its target transcripts, substantiating RBM42 role in modulating DNA damage-induced translation (Fig. 8f).

**Fig. 9:**
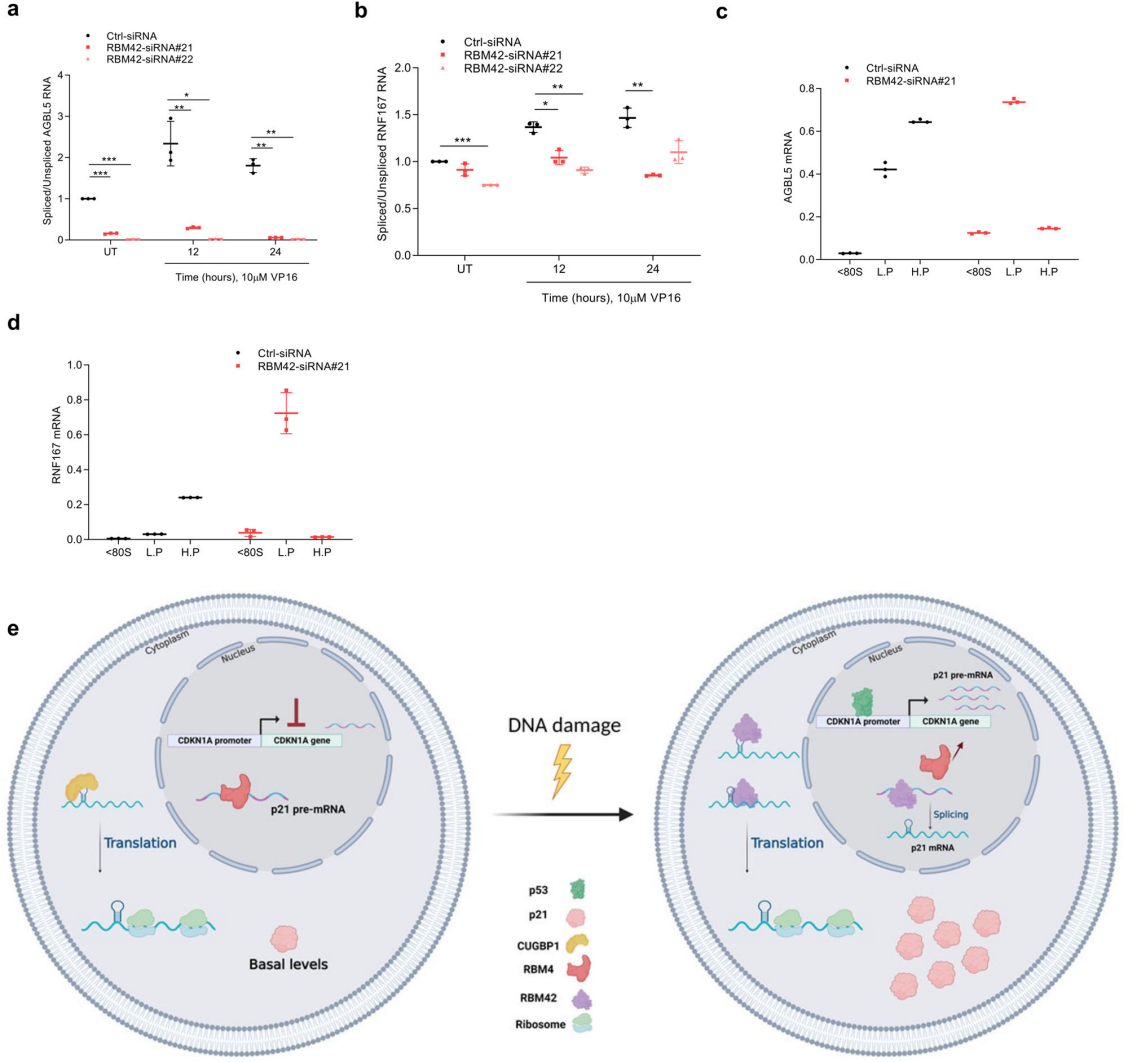
RBM42 regulates the translation of its splicing targets. **a**, **b** qRT-PCR analysis shows that RBM42 depletion disrupts the splicing of its direct splicing targets AGBL5 (**a**) and RNF167 (b). **c**, **d** RNA abundance of AGBL5 (**c**) and RNF167 (**d**) in polysomal fractions, as in Fig. 6c. in (**a**–**d**) Data are presented as mean ± s.d. (*n* = 3 biologically independent experiments). *p < 0.05, **p < 0.01, ***p < 0.001. Source data are provided as a Source Data file. **e** A model describing RBM42 dual role in finetuning splicing and translation of CDKN1A. Following DNA damage RBM42 promotes CDKN1A splicing by counteracting RBM4 binding to CDKN1A pre-mRNA. In the cytoplasm, RBM42 promotes CDKN1A mRNA translation and enabled proper induction of p21 protein levels, which is critical to maintain genome stability. Created with BioRender.com. Source data are provided as a Source Data file.

Interestingly, 40% of RBM42 direct splicing targets are also bound by RBM42 at their 5’UTR (Fig. 8e). We assumed therefore that RBM42 regulates both the splicing and translation of these genes, similar to CDKN1A. To test this assumption, we determined the effect of RBM42 depletion on the splicing and translation of two high-scoring direct splicing targets: AGBL5 and RNF167. Results confirmed that RBM42 depletion alters the splicing and the translation of these genes (Fig. 9a-d). Altogether, our data favors a model suggesting that RBM42 not only regulates the splicing of its direct target genes, but also modulates their translational efficiency during DDR (Fig. 9e).

## Discussion

Herein, we performed genome-wide multi-omics profiling of human RBM42, which revealed a previously unrecognized dual role of RBM42 in regulating splicing and translation processes during DDR. Specifically, we showed that RBM42 promotes CDKN1A splicing and translation upon DNA damage induction. Moreover, we found that RBM42 regulates the translation of additional two splicing targets (Fig. 9a-d), and therefore providing supporting evidence for a widespread coupling between splicing and translation machineries mediated by the same RNA processing factor, RBM42.

Similar to RBM42, a previous report identified a dual function of serine/arginine-rich splicing factor 3 (SRSF3) in regulating splicing and translation of PDCD4 gene^46^. Moreover, several studies demonstrated a dual role of various RNA processing factors, such as the serine/arginine-rich splicing factor 1 (SRSF1), in splicing and translation regulation. For example, it was shown that SRSF1 promotes mRNA translation by suppressing the activity of 4E-BP, a competitive inhibitor of cap-dependent translation^47–53^. Collectively, our data favor a model where the same RNA processing factors are involved in regulating multiple post-transcriptional activities to ensure exquisite finetuning of gene expression^54^.

Our data show that RBM42 binds CDKN1A RNA (Fig. 3g-j) and promotes its splicing by counteracting the binding of the negative splicing regulator, RBM4, to CDKN1A transcript (Fig. 5). Beside RBM42, a previous report identified SKIP as a selective splicing regulator of CDKN1A pre-mRNA^16^. Future work will be required to investigate a potential crosstalk between RBM42 and SKIP in regulating CDKN1A splicing. In addition, it would be important to address whether, similar to RBM42, SKIP also regulates the translation of its splicing targets.

RBM42 interactome analysis revealed that it is associated with multiple translational factors including the eukaryotic translation initiation factor 2 subunit 1 (eIF2a), which undergoes phosphorylation by integrated stress response (ISR) kinase, GCN2, following stress conditions leading to CDKN1A translation initiation^22,55^. Additionally, interactome analysis revealed proximity between RBM42 and CUGBP1, which was shown to regulate CDKN1A translation in the absence of DNA damage^17,42^. Interestingly, our data show that the same SL sequence within the 5’ region of CDKN1A mRNA is required for RBM42 and CUGBP1 binding to CDKN1A transcript. However, contrary to CUGBP1, RBM42 predominantly binds CDKN1A mRNA after DNA damage induction. These results suggest that while CUGBP1 is required for basal translation of CDKN1A, RBM42 promotes CDKN1A translation following DNA damage induction. It remains unknown however what governs the binding affinity between RBM42 and CUGBP1 to CDKN1A mRNA, and whether RBM42 and CUGBP1 directly bind the SL region of CDKN1A transcript. Notably, our data implicating RBM42 in translation regulation is in line with a recent report showing that SRSF1 and RBM42 bind to specific 5′UTR sequences to modulate the translation of a subset of c-Myc target genes^29,56^.

Beside the effect of RBM42 on p21 translation, the polysomic profile and OPP assay showed a global effect of RBM42 on protein translation (Fig. 6b, c). Accordingly, RBM42 interactome and eCLIP data suggest that RBM42 may facilitate global translation through its interaction with translation factors (Fig. 4f) and binding to 5’UTR (Fig. 8f). In addition, RBM42 depletion leads to misregulation of genes annotated as ribosome-associate genes (Fig. 1c), raising a possibility that misregulation of these genes contributes to the decrease in translation seen in RBM42-deficient cells.

Accumulating evidence implicate RNA processing and splicing factors in DDR. Some of these splicing factors promote the generation of DNA damage-specific transcripts that are essential for intact repair of DNA lesions^57–72^. In agreement with this, we found that the expression and the splicing of hundreds of genes are altered in RBM42-dependent manner after DNA damage (Fig. 1; 3). Pathway enrichment analysis revealed that a substantial number of genes regulated by RBM42 during DNA damage are implicated in regulating cell cycle progression, apoptosis, and DNA repair. Our data therefore identified RBM42 as a regulator of genome stability. Notably, RBM42 is predominantly overexpressed in a variety of human cancers^73^. Future studies will be required therefore to determine whether RBM42 plays a role in carcinogenesis that is dependent on its regulatory effect on p21.

## Methods

### Plasmids

pEGFP-N1-RBM42, p3x-Flag-CMV10-RBM42, p3x-Flag-CMV10-RBM42-ΔRRM, p3x-Flag-CMV10-p21^WT^, p3x-Flag-CMV10-p21^ΔSL^, pCDNA3.1 Hygro-6xmyc-CUGBP1, pCDNA3.1 Hygro-6xmyc-RBM4, pBluescript II-KS-( + )-RBM42-LHA-Neo-RHA, pGEX4T3-RBM42, pLKO.1-TRC-RBM4-shRNA were constructed as described in Supplementary Data 7. Complete list of all primers and their sequences is described in Supplementary Data 7. pSpCAS9 (BB)−2A-GFP (PX458; #48183) and pspgRNA (#47108) vectors were purchased from Addgene. All constructs used in this study were verified by nucleotide sequencing or restriction digestion.

### Cell lines

All cell lines used in this study were obtained from ATCC. All cell lines used were cultured in media supplemented with 10% heat-inactivated FBS, 2mM L-glutamine (Gibco), 100 unit/mL penicillin and 100 μg/mL streptomycin (Gibco). U2OS (ATCC, HTB-96) and HEK293T (ATCC, CRL-3216) cell lines were cultured in Dulbecco’s modified Eagle’s medium (Gibco). HCT116 (ATCC, CCL-247) cell line was cultured in RPMI-1640 media (Gibco).

### Transfections and drug treatments

Cell transfections with plasmid DNA or siRNA were performed using Polyethylenimine (PEI) and Lipofectamine RNAiMax, respectively, following the manufacturer’s instructions.

### Western blot

Protein extracts were prepared using Hot-lysis buffer (1% SDS, 5 mM EDTA, 50 mM Tris, pH 7.5 and protease inhibitor mixture (Calbiochem)). Samples were separated on SDS-PAGE gel and membranes were immunoblotted with the relevant antibodies (a complete list of antibodies and their dilutions is described in Supplementay Data 9).

### Immunofluorescence

Cells were grown on coverslips for 24 h and subjected to immunofluorescence as previously described^64^. Cells were immunostained with RBM42 antibody (Supplementay Data 9). Slides were visualized using the inverted Zeiss LSM-700 confocal microscope with 40× oil EC Plan Neofluar objective.

### Chromatin immunoprecipitation (ChIP)

ChIP was performed as previously described^60,74^. Briefly, control and RBM42-depleted HCT116 cells were plated in 150 mm dishes. 72 hours following siRNA transfection cells were exposed to ionizing radiation (5 Gy) or left untreated, and 4 hours later were crosslinked with 1% PFA for 10 min at room temperature. Crosslinking was stopped with 0.125 M Glycine for 5 min. After scraping and cell lysis, DNA was sheared to the size of 300–500 bp using a Vibra cell sonicator (15 sec ON, 30 sec OFF, 38% duty, 20 cycles). 5% of each supernatant was used as input control. The rest of the supernatant was subjected to overnight immunoprecipitation (IP) using 1 μg p53 (DO-1 santa cruze) and protein A magnetic beads (GenScript). Following reverse cross-linking; the precipitated DNA was purified using the PureLinkTM PCR Micro Kit. Quantification of the immunoprecipitated DNA was carried out by Step-One-Plus real-time PCR using Fast SYBR Green Master mix (Applied Biosystems) and the primers around two known p53 binding sites located at p21 DNA (Supplementary Data 8).

Fold induction was calculated and values were normalized to the no-antibody control (IgG).

For RBM42 ChIP we used HCT116^RBM42-Flag^ Cell line and Flag antibody.

### Cross-linking and immunoprecipitation (CLIP)-qRT-PCR

HCT116^RBM42-Flag^ and HCT116 cells were treated with 20 µM VP16 or left untreated. 18 hours later cells were UV cross-linked (400mJ/cm^2^ at 254 nm) and scrapped. Cells were lysed with RIPA buffer (150 mM NaCl, 1% NP-40, 0.5% Deoxycholate, 0.1% SDS, 50 mM Tris, 5 mM EDTA, RNase inhibitor and protease inhibitors) and sonicated (15 sec ON, 35% duty, one cycle). 5% of each supernatant was used as input control. The rest of the supernatant was subjected to overnight immunoprecipitation (IP) using Flag antibody and protein G magnetic beads (GenScript). Beads were washed, treated with RQ1 DNaseI for 10 min and with Proteinase K for 15 min. TRIzol reagent was added to the elutes and RNA was extracted. Quantification of the immunoprecipitated RNA was carried out by Step-One-Plus real-time PCR using Fast SYBR Green Master mix (Applied Biosystems) and primers amplifying p21 mRNA (Supplementary Data 8).

### eCLIP

Untreated and VP16-treated cells (20 µM VP16 for 18 hours), two biological replicates each, were UV cross-linked (400mJ/cm2 at 254 nm). Cells were lysed and sonicated using Covaris E220 for two minutes using the following settings: intensity 140, burst 200, and duty 5. RNA was cleaved by 40 units of RNase I for 3 minutes at 37 °C. Lysates were cleared by centrifugation for 10 minutes and 50 µl protein-G beads for 30 minutes. Cleared lysates were immunoprecipitated using 3 µg RBM42 antibody for 4 hours followed by 1.5 hours incubation with 50 µl protein-G beads. Beads were washed, then underwent end repair and 3’ adapter ligation. The protein-RNA complexes were eluted, resolved on a polyacrylamide gel, and then transferred onto a nitrocellulose membrane. RNA was extracted from the membrane using incubation in 0.2% SDS buffer containing 32 units of proteinase K at 50 °C for 1 hour. RNA purification and library preparation were carried out as in the original protocol^75–77^, except for cDNA synthesis, which was performed using SuperScript III reverse transcriptase.

eCLIP reads were processed as in the ENCODE eCLIP-seq processing pipeline (https://github.com/YeoLab/eclip/blob/master/documentation/eCLIP_single_end_analysisSOP_v1.docx) using GRCh38 genome version on the Galaxy platform (https://usegalaxy.eu). Peaks were called for each treatment separately using PureCLIP^75^. UMI collapsed reads were counted to the peaks. Read counts were normalized, and differential enrichment between the eCLIP and their inputs was calculated by the NOISeq R package, using the “noiseq” function. Peaks annotation and further analysis were performed using the RCAS package in R.

### RNA Isolation, reverse transcription, and quantitative real‐time PCR

Total RNA was extracted from cells using TRIzol reagent according to the manufacturer’s instructions (Ambion). 1 μg RNA was used for cDNA synthesis using the qScript cDNA Synthesis Kit (Quanta) with random primers. mRNA levels were measured by real‐time PCR in the Step‐One‐Plus real‐time PCR System (Applied Biosystems) using Fast SYBR Green Master mix (Applied Biosystems) with three technical repeats for each PCR with the indicates primers. Data analysis and quantification were performed using StepOne software V2.2 supplied by Applied Biosystems. GAPDH gene was used as a housekeeping gene.

### Tagging of the endogenous RBM42 protein using CRISPR-Cas9

CRISPR-Cas9 knock-in technique was used to fuse either 3xFlag, GFP or 3xFlag-APEX2 at the C terminus of the endogenous RBM42 gene. HCT116 cells were co-transfected with px330 plasmid containing Cas9 nuclease and gRNA targeting the end of RBM42 coding sequence (upstream RBM42 stop codon) and a donor plasmid containing neomycin, P2A self-cleavage site and the relevant tag sequence (e.g 3XFlag) flanked by homology arms (∼700 bp each side) corresponding to RBM42 gene. Neomycin-resistant clones were screened by western blot and genomic PCR using the primer sequences provided in Supplementary Data 8.

### APEX-based proximity labeling and affinity enrichment of biotinylated proteins and preparation for MS analysis

Positive HCT116^APEX2-RBM42^ clones were validated by western blot and by monitoring APEX2-dependent protein biotinylation in the engineered cell lines. Untreated and VP16 treated control HCT116 and HCT116^APEX2-RBM42^ cells were subjected to APEX-based proximity labeling assay as previously described^58^. Peptides were injected into mass spectrometry (MS) at the Smoler Proteomics Center in the department of Biology in the Technion. Samples were analyzed by LC-MS/MS using Q-Exactive plus mass spectrometer (Thermo Scientific), coupled to Easy nano LC-1000 capillary UHPLC (Thermo Scientific) as described in^58^. The resulted tryptic peptides from on-beads digestions were resolved by a reverse phase chromatography using homemade fused silica capillary (0.075x200mm) packed with Reprosil reversed phase material (Dr Maisch GmbH), in 0.1% formic acid. The peptides were eluted with a 120 min linear gradient of 5% to 28% acetonitrile with 0.1% formic acid (in water), followed by 15 min linear gradient 28% to 95%, and 10 min at 95% acetonitrile with 0.1% formic acid at flow rates of 0.15 µl/min. Mass-spectrometry was performed with data-dependent acquisition mode for positive ions at mass range of 300–1800 m/z with resolution of 7000, selecting the 10 most intense ions (with charge>1) in each full MS. Ions fragmentation in MS/MS was done by high collision-induced dissociation (HCD) at 25 normalized collision energy. The AGC was set to 3 × 10^6^ for the full MS and to 1 × 10^5^ for the MS/MS scans. The intensity threshold for triggering MS/MS was set 1 × 10^4^, and the dynamic exclusion duration was set to 20 sec. The MS data was analyzed using MaxQuant^78^ version 1.6.5.0, searching against the human protein of the Uniprot database (download date: 1/7/2019), using the default settings and including LFQ and match-between-runs options. Statistical analysis was done using Perseus software platform (1.6.5) ^79^and proteins with difference in LFQ intensity > 2 and p-value < 0.05 between HCT116^RBM42-APEX2^ and control cells were classified as significantly enriched. Proteins significantly enriched in APEX2-RBM42-expressing cells were subsequently analyzed for pathway enrichment using ShinyGO^80^.

### Polysome profiling

U2OS cells were transfected with Ctrl or RBM42 siRNA. 48 hours following siRNA transfection cells were treated with 5 µM VP16 for 18 hours. Next, cells were incubated for 1 min with 0.1 mg/ml cycloheximide (CHX) to stabilize ribosomes. Cells were immediately harvested by scraping with ice-cold PBS supplemented with 0.1 mg/ml CHX and centrifuged. Cell pellet was supplemented with lysis buffer (20 mM Tris pH 7.4, 140 mM KCl, 1.5 mM MgCl_2_, 0.5 mM DTT, 0.1 mg/ml CHX, 1% Triton, 40U/ml RNase inhibitor, protease inhibitors (1:500)), and incubated on ice for 5 min. 10–50% linear sucrose gradients were prepared (50 mM Tris-HCl (pH = 7.5), 50 mM NH4Cl, 12 mM MgCl2, 0.5 mM DTT, 100 μg/ml CHX). RNA content in each lysate was measured, and equal amounts of RNA from each sample (800 μg) were loaded on the sucrose gradients and centrifuged in a Beckman SW41 rotor at 273000 g and 4 °C for 2 h. Gradients were fractionated, fractions were used for phenol-chloroform RNA extraction and analyzed by RT-PCR.

### Protein purification

RBM42 was cloned into pGEX-6P-3 vector. Empty pGEX-6P-3 and pGEX-6P-3-RBM42 were transformed into BL21 bacteria and grown in 2XTY+Amp medium at 37 °C up to O.D 0.4-0.6. bacteria were incubated with 0.1Mm IPTG at 18 °C overnight to induce protein expression. Cells were centrifuged and pellet was lysed (PBSx1, 15 mM EDTA and 1:200 PMSF and PI) and homogenized. Cells were disrupted using homogenizer (Polytran). samples were incubated in rotation with 0.5% NP-40 and 5 mM DTT for 30 min followed by centrifugation. Glutathione beads were washed with ice-cold PBSx1 + 5Mm DTT. Lysate was incubated with the beads overnight at 4 °C. samples were run on acrylamide gel and the gel was stained with Coomassie.

### RNA in vitro binding

Purified GST or GST-RBM42 fused to glutathione beads were incubates with total RNA extracted from HCT116 cells for 4 hours. Beads were washed (50 mM Tris-HCl (pH 7.4), 0.5 M NaCl, 1 mM EDTA, 1% NP-40, 0.5% Deoxycholate, RNase inhibitor) buffer and RNA was extracted from the beads using TRizol reagent. RNA eluted from the beads was quantified using qRT-PCR with primers for p21 and GAPDH.

### Cell cycle analysis by flow cytometry

Flow cytometric analysis was performed as previously described (Khoury-Haddad et al., 2014). Briefly, cells were fixed with ice-cold 75% ethanol. DNA was stained with 100 mg/ml propidium iodide (Sigma-Aldrich) in phosphate buffer solution (PBS) containing 0.1% Triton-X-100 and 0.5 mg/ml DNase-free RNase A (Sigma-Aldrich). Samples were analyzed using flow cytometry of 10,000 events on a BD LSR-II flow cytometer (Becton Dickinson). Data were analyzed with FCS express software.

### Short-term growth delay assay

For determining drug sensitivity, cells were seeded in 96-well plates in triplicates at a density of 5,000 cells per well. 24 h postseeding, drugs were added at the indicated concentrations. Cell viability was measured 72 h after drug treatment using the CellTiter 96® AQueous One Solution Cell Proliferation Assay (Promega) following the manufacturer’s protocol, and absorbance was measured using Epoch Microplate Spectrophotometer (BioTek). Cell viability was normalized to the viability of untreated cells.

### Cell viability testing with Trypan blue

Cells were seeded in 12-well plates in triplicates at a density of 500 cells per well. Cells were stained with Trypan-blue following the manufacturer’s protocol, and cell viability was calculated using CellDrop (DeNovix Inc).

### RNA-sequencing

Three biological replicates of RNA samples were purified from HCT116 cells transfected with control or RBM42 siRNA#21 and treated with 20 μM VP16 for 12 h. RNA sequencing libraries were prepared using TruSeq mRNA library preparation kit. Sequencing was performed at The Crown Genomics institute of the Nancy and Stephen Grand Israel National Center for Personalized Medicine,Weizmann Institute of Science using a NovaSeq 6000 system with S1 flow cell to obtain 150 bp paired-end reads. Average read depth was 60 million reads per sample. The quality of the raw FASTQ files was assessed using FastQC software (http://www.bioinformatics.babraham.ac.uk/projects/fastqc/). For differential gene expression analysis, raw sequencing reads were aligned to GENCODE GRCh38 genome assembly using Salmon package^81^ and differential gene analysis was performed in R using the DESeq2 package^82^. To analyze alternative splicing events, raw reads were mapped to GENCODE GRCh38 genome assembly using the splice-sensitive aligner HISAT2^83^. Alternative splicing events were detected using rMATS package^84^. Differential exon expression analysis was performed in R using DEXSeq package^85^. Pathway enrichment analysis and gene ontology was conducted using ShinyGO^80^. Coverage tracks were visualized using Integrated Genome Viewer (IGV)^86^.

### Annexin V assay

Apoptosis was assessed by annexin V-FITC (BioVision, 1006-200) according to the manufacturer’s instructions. HCT116 cells were transfected with RBM42 or control siRNA, treated with 3 Gy IR and recovered for 12 and 24 h. Samples were analyzed using flow cytometry of 10,000 cells on a BD™ LSR II flow cytometer (BD Biosciences) and analyzed by BD FACSDiva™ software, version 6.1.2. (BD Biosciences). Results were calculated as the percentage of positive annexin V-FITC cells out of total cells counted.

### Minigene splicing reporter assay

RBM42 minigene splicing reporter (pcDNA3.1-CDKN1A-SR-ex2-3) was constructed as shown in Supplementary Data 7. U2OS cells were transfected with control and RBM42 siRNA. Minigene reporter (1000 ng) was transfected into the cells 36 h after siRNA transfection. Next, cells were treated with 10μM VP16 for 12 h and harvested 72 h after siRNA transfection. Total RNA was extracted using TRIzol reagent, followed by cDNA synthesis using qScript cDNA Synthesis Kit (Quanta) according to the manufacturer’s instructions. Samples were then subjected to semiquantitative PCR analysis using the indicated primers in Supplementary Fig. 4g. PCR product were run in agarose gel and quantified using Gel Doc software (BioRad).

### 3’Rapid amplification of cDNA end (RACE) assay

HCT116 cells were transfected with siRNA against RBM42 or control siRNA. Total RNA was extracted using TRIzol reagent and cDNA was synthesized using qScript cDNA Synthesis ultra-flex Kit (Quanta) and Oligo d(T)-Anchor primer (Supplementary Data 7), according to the manufacturer’s instructions. Samples were then subjected to PCR analysis. The 3’RACE was also performed on U2OS cells transfected with CDKN1A minigene using the indicated primers (Supplementary Fig. 4h-i)

### OPP assay

The OPP assay (OPP, Thermo Fisher Scientific) was carried out following the manufacturer’s instructions, as described in ref. ^87^. In brief, U2OS cells were transfected with control of RBM42 siRNAs. Cells were seeded on coverslips and treated with 5μM VP16 for 18hrs. 72hrs after siRNA transfection cells were treated with 20 μM OPP for 1 hr at 37 °C. The cells were then fixed for 15 min using 1% formaldehyde, and permeabilized using 0.15% Triton X-100 and 0.15% TWEEN 20 in PBS for 15 min. Cells were then incubated for 30 min at room temperature with Click reaction buffer containing 100 mM Ascorbate, 2 mM CuSO4, and 1 μM 647-azide (Thermo Fisher Scientific) in PBS to visualize OPP. Cells were counterstained with DAPI and coverslips were mounted on glass microscope slides and imaged using confocal microscopy. Signal intensity was quantified using ImageJ software.

### Statistical analysis

Statistical analyses were performed using GraphPad Prism 8 software. Statistical parameters are expressed as the mean ± SD and corresponding sample size and *P* values are reported in the Figures and Figure legends. Statistical analysis between two groups were done by paired or unpaired and two-tailed *t*-test.

### Reporting summary

Further information on research design is available in the Nature Portfolio Reporting Summary linked to this article.

### Supplementary information

Supplementary Information

Description of Additional Supplementary Files

Supplementary Data 1

Supplementary Data 2

Supplementary Data 3

Supplementary Data 4

Supplementary Data 5

Supplementary Data 6

Supplementay Data 7

Supplementary Data 8

Supplementary Data 9

Reporting Summary

### Source data

Source Data

## Data Availability

Raw RNA-seq data has been deposited at ArrayExpress with accession number E-MTAB-11877. The mass spectrometry data has been deposited to the ProteomeXchange Consortium via the PRIDE partner repository with the dataset identifier PXD034854. Raw eCLIP sequencing data has been deposited in NCBI’s Gene Expression Omnibus and are accessible through GEO Series accession number GSE245744. All data supporting the findings of this study are available from the corresponding author upon request. Source data are provided with this paper.
