# Supplementary material for: A dual role of RBM42 in modulating splicing and translation of CDKN1A/p21 during DNA damage response
Source: Nat Commun. 2023 Nov 22;14:7628. doi: 10.1038/s41467-023-43495-6 (PMC10665399; doi:10.1038/s41467-023-43495-6)
Supplement: Supplementary file 1 — Supplementary Information [file 41467_2023_43495_MOESM1_ESM.pdf]

Supplemental information

A dual role of RBM42 in modulating splicing and translation of CDKN1A/p21 during DNA damage response

Bella M. Ben-Oz, Feras E. Machour, Marian Nicola, Amir Argoetti, Galia Polyak, Rawad Hanna, Oded Kleifeld, Yael Mandel-Gutfreund and Nabieh Ayoub[1]

Department of Biology, Technion - Israel Institute of Technology, Haifa 3200003, Israel
[1]Corresponding author: ayoubn@technion.ac.il

This file includes supplementary figures 1-8

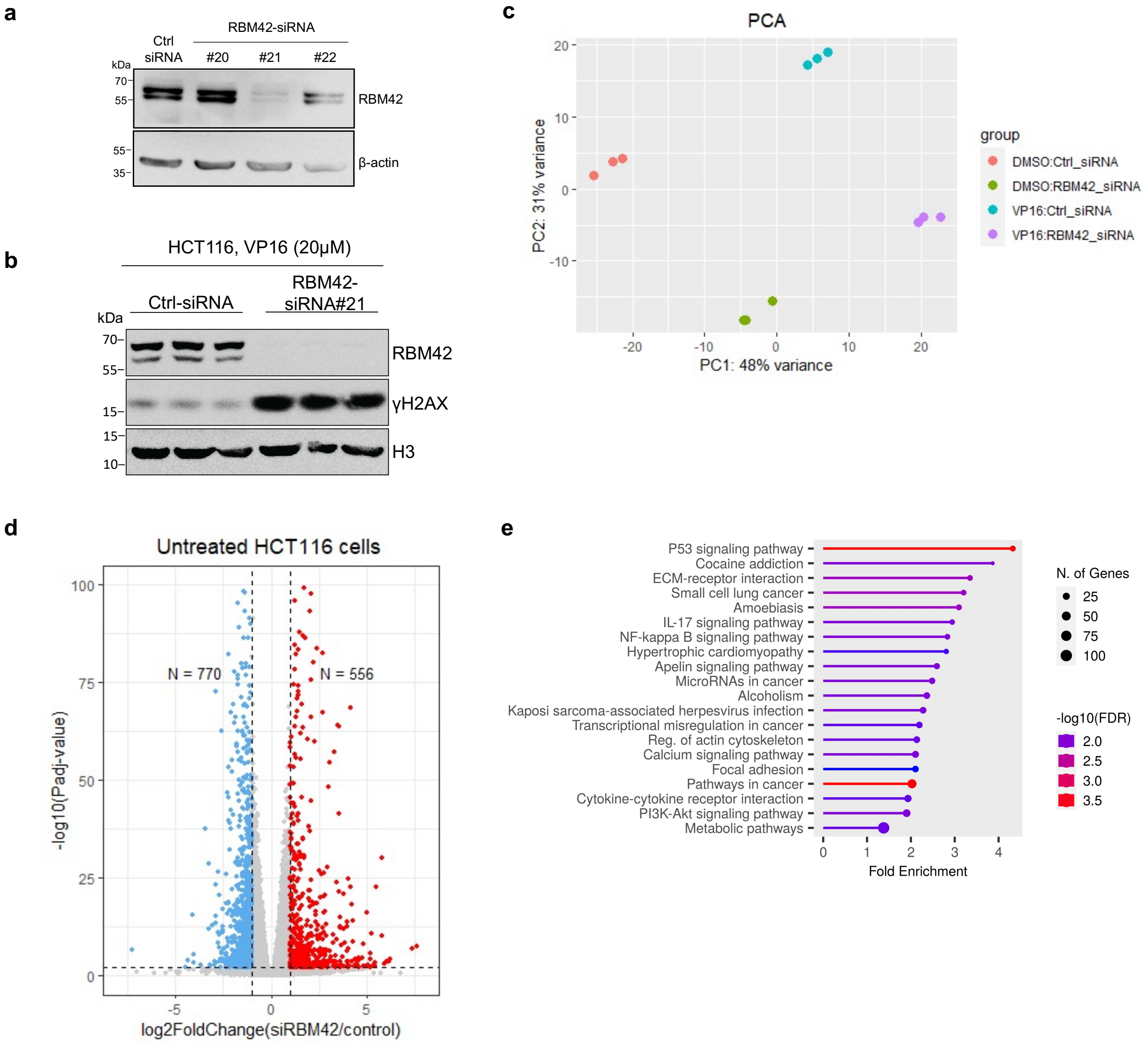

Supplementary Figure 1. Related to Fig. 1: **(a)** Western blots shows the knockdown efficiency of 3 RBM42 siRNA sequences. **(b)** Western blot shows RBM42 knockdown using siRNA#21 in 3 biological replicates of HCT116 treated with 20μM VP16 for 18h. **(c)** Principal component analysis (PCA) of RNA-seq data of HCT116 cells shows a high degree of reproducibility among the replicate samples within each group and significant variance between the different groups. **(d)** Volcano plot summarizing differential gene expression data obtained from RNA-seq analysis between control and siRBM42-transfected HCT116 cells. Upregulated genes with log2FoldChange(siRBM42/control) > 1 and Padj-value < 0.01 are marked in red, while downregulated genes with log2FoldChange(siRBM42/control) < -1 and Padj-value < 0.01 are marked in blue. N indicates the number of significantly upregulated or downregulated genes. **(e)** KEGG pathway enrichment analysis of differentially expressed genes obtained from RNA-seq data between control and siRBM42-transfected HCT116 cells. Source data are provided as a Source Data file.

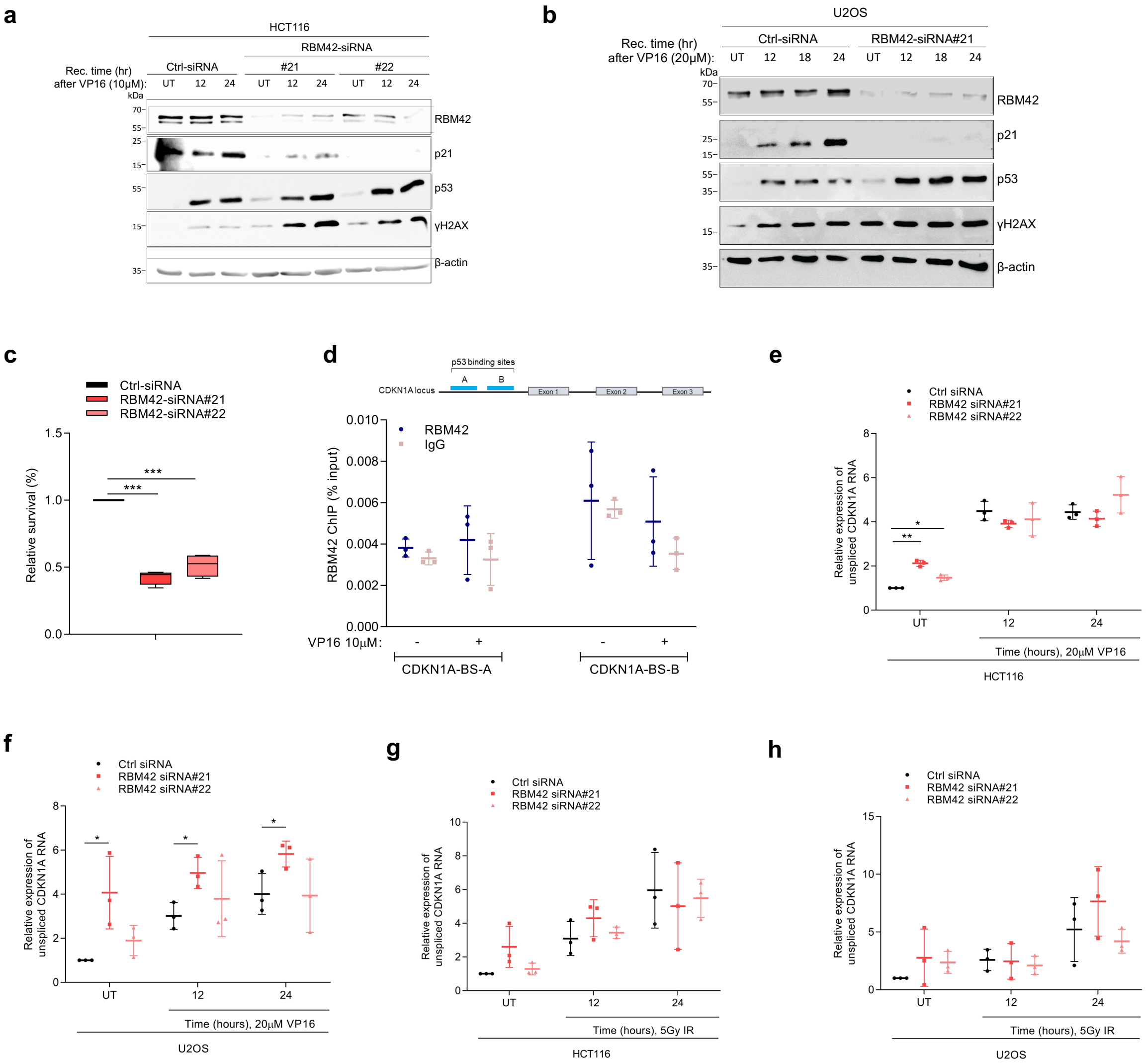

Supplementary Figure 2. Related to Fig. 2: **(a-b)** Western blot analysis, as in Figure 2a, shows that RBM42 depletion impairs the DNA damage-induced increase of p21 protein levels in HCT116 (a) and U2OS (b) cells. **(c)** RBM42 depletion decreases cell viability. Short-term cell viability assay in RBM42-proficient and -deficient HCT116 cells. P-*value*(si#21) = 0.0002; P-*value*(si#22) = 0.001 **(d)** RBM42 ChIP-qPCR shows that RBM42 is not recruited to CDKN1A promoter before and after VP16 treatment. HCT116 cells were left untreated (UT) or treated with 10μM VP16 for 4h. Cells were subjected to ChIP-qPCR using RBM42 antibody. (Top) Schematic representation of two p53 binding sites (BS-A and BS-B) within p21 locus recognized by qPCR primers. **(e-h)** RBM42 depletion has no significant effect on the levels of CDKN1A nascent transcript during DNA damage in HCT116 (e, g) and U2OS (f, h) cells treated with VP16 or IR, respectively. RT-qPCR analysis was performed as described in Figure 2h. (e) P-*value*(si#21, UT) = 0.004; P-*value*(si#22, UT) = 0.02. (f) P-*value*(si#21, UT) = 0.03; P-*value*(si#21, 12h) = 0.02; P-*value*(si#21, 24h) = 0.04. Data are presented as mean ± s.d. (n=3 biologically independent experiments). All the statistical tests are two-tailed, tow-sided *t-test*. *p < 0.05, **p < 0.01, ***p < 0.001, ****p < 0.0001. Source data are provided as a Source Data file.

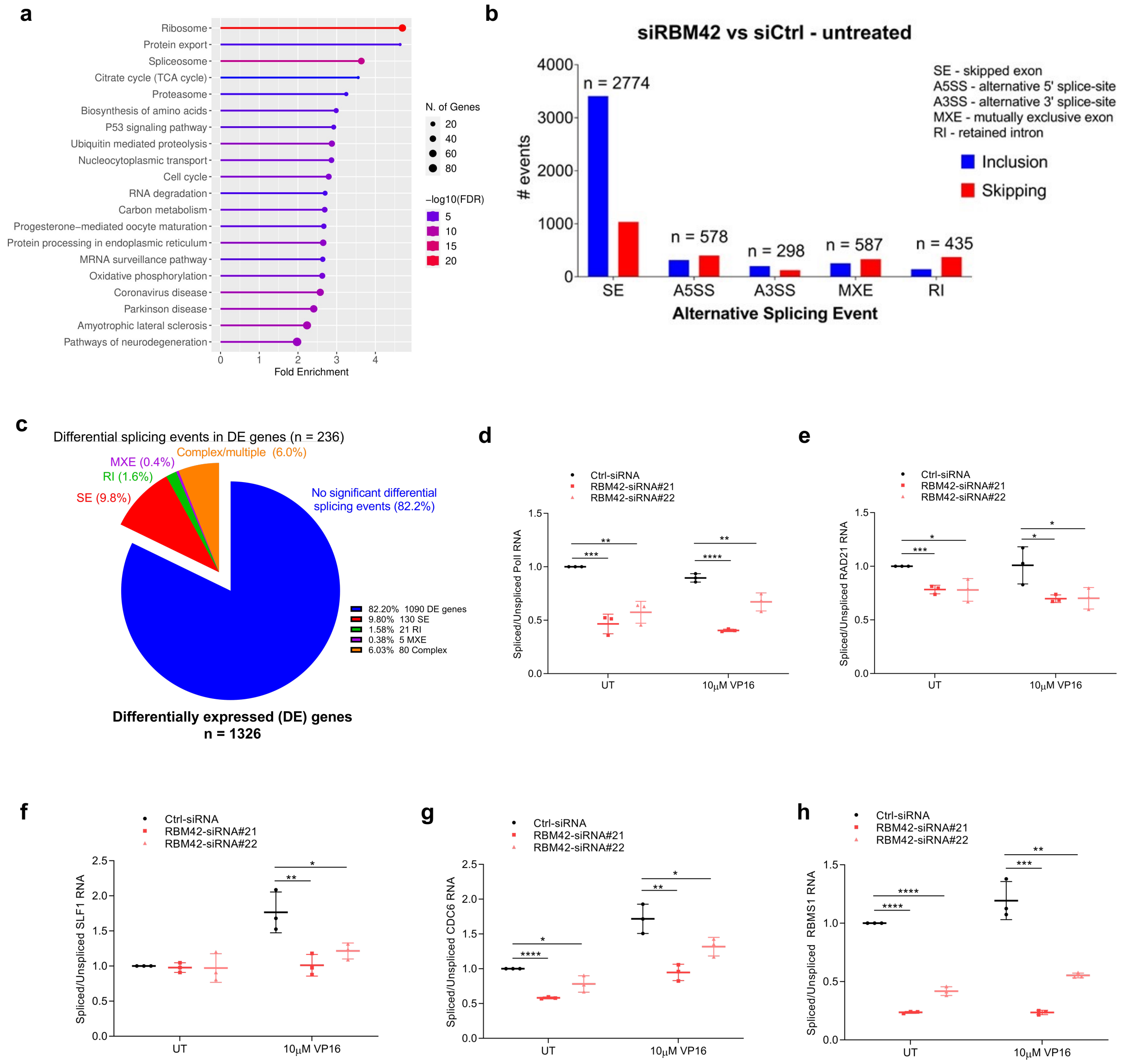

Supplementary Figure 3. Related to Fig. 3: **(a)** Gene set enrichment analysis (KEGG pathways) of RBM42 splicing targets from RNA-seq data between control and RBM42-deficient HCT116 treated with 20µM VP16 for 18h. **(b)** Summary of significant alternative splicing events (leading to skipping (red) or inclusion (blue) observed upon RBM42 depletion in untreated (UT) HCT116 cells as detected by rMATS[38]. Significantly altered splicing events were classified as having a minimum inclusion level difference of 0.1, p-value<0.01, and FDR<0.01. SE: skipped exon, MXE: mutually exclusive exons, A5SS: alternative 5' splice site, A3SS: alternative 3' splice site, IR: intron retention. **(c)** Pie chart showing the distribution of significant alternative splicing events among the differentially expressed genes upon RBM42 depletion in UT cells. **(d-h)** RBM42 knockdown disrupts the splicing of its target genes identified by RNAseq. RT-qPCR analysis was performed as described in Figure 3f. (d) P-*value*(si#21, UT) = 0.0005; P-*value*(si#22, UT) = 0.002; P-*value*(si#21, VP16) = 0.00003; P-*value*(si#22, VP16) = 0.01. (e) P-*value*(si#21, UT) = 0.0007; P-*value*(si#22, UT) = 0.02; P-*value*(si#21, VP16) = 0.03; P-*value*(si#22, VP16) = 0.05. (f) P-*value*(si#21, VP16) = 0.01; P-*value*(si#22, VP16) = 0.03. (g) P-*value*(si#21, UT) = 0.0000007; P-

value(si#22, UT) = 0.03; P-*value*(si#21, VP16) = 0.005; P-*value*(si#22, VP16) = 0.05. (h) P-*value*(si#21, UT) = 0.000000008; P-*value*(si#22, UT) = 0.00001; P-*value*(si#21, VP16) = 0.0005; P-*value*(si#22, VP16) = 0.002. Data are presented as mean ± s.d. (n=3 biologically independent experiments). The statistical tests in (d-h) are two-tailed, tow-sided *t-test*. *p < 0.05, **p < 0.01, ***p < 0.001, ****p < 0.0001. Source data are provided as a Source Data file.

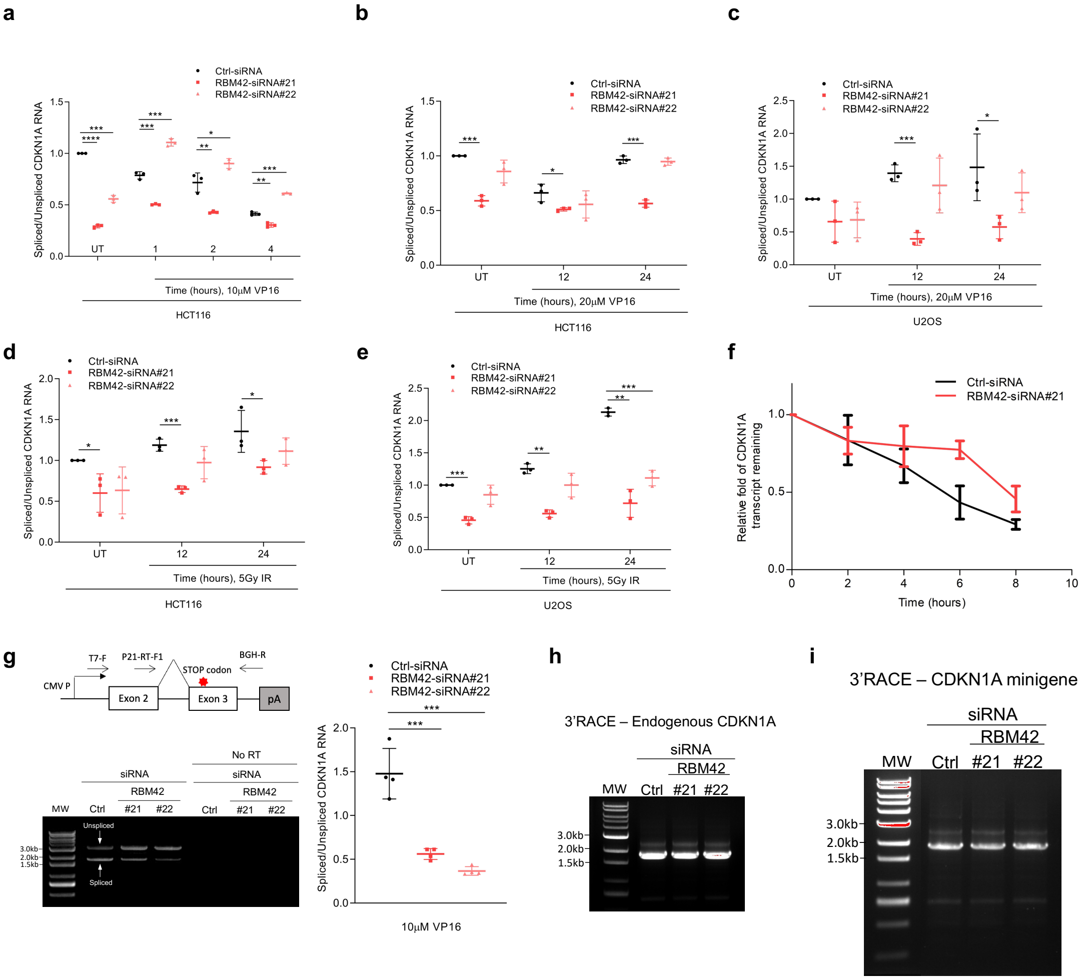

Supplementary Figure 4. Related to Fig. 3: **(a-e)** RBM42 knockdown disrupts CDKN1A splicing in HCT116 and U2OS cells treated with VP16 (a-c) and IR (d-e). RT-qPCR analysis was performed as described in Figure 3f. (a) P-*value*(si#21, UT)=0.0000002; P-*value*(si#22, UT)=0.00002; P-*value*(si#21, 1h)=0.0002; P-*value*(si#22, 1h)=0.0004; P-*value*(si#21, 2h)=0.006; P-*value*(si#22, 2h)=0.04; P-*value*(si#21, 4h)=0.002; P-*value*(si#22, 4h)=0.00004. (b) P-*value*(si#21, UT)=0.0001; P-*value*(si#21, 12h)=0.03; P-*value*(si#21, 24h)=0.0001. (c) P-*value*(si#21, 12h)=0.0004; P-*value*(si#21, 24h)=0.04. (d) P-*value*(si#21, UT)=0.04; P-*value*(si#21, 12h)=0.0004; P-*value*(si#21, 24h)=0.04. (e) P-*value*(si#21, UT)=0.00008; P-*value*(si#21, 12h)=0.0002; P-*value*(si#21, 24h)=0.0004; P-*value*(si#22, 24h)=0.0001.**(f)** qRT-PCR shows that RBM42 depletion doesn't reduce CDKN1A mRNA stability. HCT116 cells transfected with control (Ctrl) and RBM42 siRNA were treated with 10□M VP16 for 18h. The *x* axis represents time after addition of actinomycin-D (5μg/ml), and the *y* axis represents relative levels of

remaining CDKN1A mRNA in cells. **(g)** Top: schematic of CDKN1A minigene splicing reporter. Arrows indicate the locations of the PCR primers CDKN1A-specific forward primer (P21-RT-F1) and vector-specific reverse primer (BGH-R). Bottom: Representative gel image of CDKN1A minigene RT-PCR analysis in U2OS cells transfected with either control or RBM42 siRNA. No RT - no reverse transcriptase control. Right: quantification of the ratio between the spliced and unspliced CDKN1A variants amplified from the minigene reporter. Data represents mean ± SD (n=4 biologically independent experiments). P-*value*(si#21)=0.0008; P-*value*(si#22)=0.0003 **(h)** 3' Rapid Amplification of cDNA Ends (RACE) of endogenous CDKN1A in HCT116 cells transfected with control (Ctrl) or RBM42 siRNA and treated with 10□M VP16 for 18h. RNA was reverse transcribed using Oligo d(T)-Anchor primer, followed by PCR amplification using reverse RACE anchor primer and CDKN1A-specific forward primer (P21-RT-F1). **(i)** as in (h), except of using U2OS cells transfected with CDKN1A minigene splicing reporter. PCR amplification was performed using reverse RACE anchor primer and minigene-specific forward primer (T7-F). Data are presented as mean ± s.d. (n=3 biologically independent experiments). The statistical tests are two-tailed, tow-sided *t-test*. *p < 0.05, **p < 0.01, ***p < 0.001, ****p < 0.0001. Source data are provided as a Source Data file.

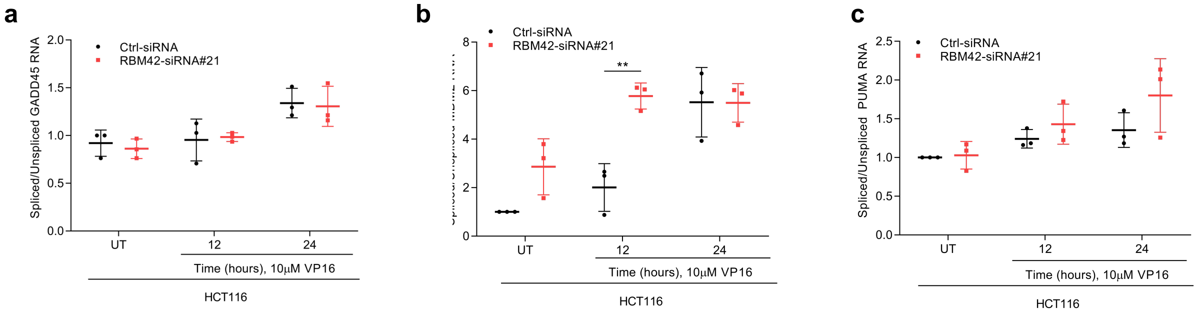

Supplementary Figure 5. (a-c) qRT-PCR shows that RBM42 depletion doesn't affect the splicing of p53 target genes GADD45 (a), MDM2 (b) and PUMA (c). RT-qPCR analysis was performed as described in Figure 3f. (b) P-*value*(12h) = 0.004. Data are presented as mean ± s.d. (n=3 biologically independent experiments). The statistical tests are two-tailed, tow-sided *t-test*. *p < 0.05, **p < 0.01, ***p < 0.001, ****p < 0.0001. Source data are provided as a Source Data file.

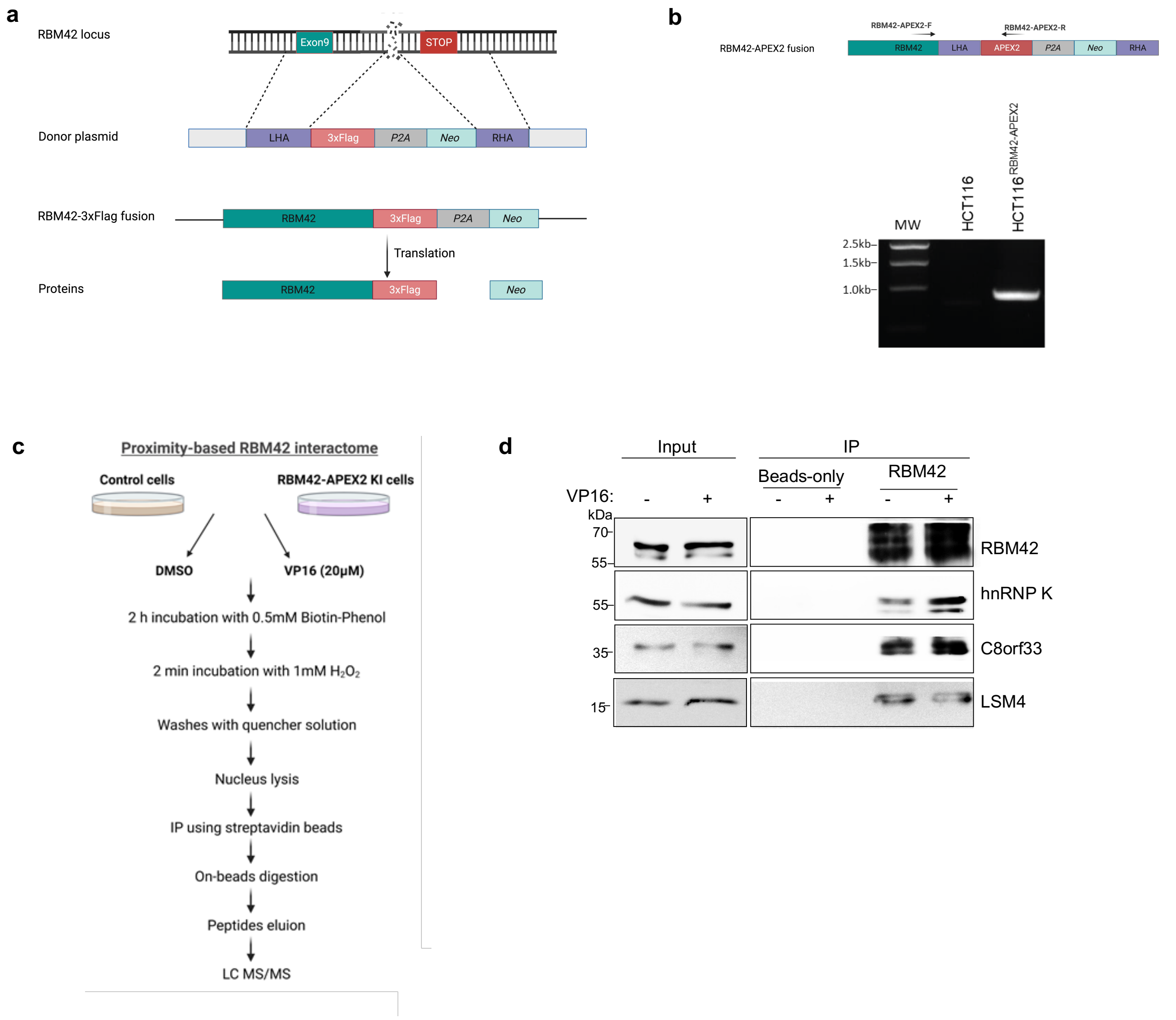

Supplementary Figure 6. Related to Figure 4. **(a)** Schematic diagram describing the strategy for CRISPR-based APEX2 knock-in at the C-terminus of RBM42 gene. Created with BioRender.com. **(b)** PCR analysis confirming the biallelic knock-in of APEX2 at the C-terminus of the endogenous RBM42 gene. A schematic diagram of primers' positions for validating APEX2-P2A-neo cassette integration at the C-terminus of RBM42 gene Created with BioRender.com (Top). **(c)** Schematic depiction of the workflow for identifying RBM42 interactome using APEX2 proximity labelling. Created with BioRender.com. **(d)** Immunoprecipitation of endogenous RBM42 in HCT116 cells shows interaction with three proximal proteins hnRNP K, C8orf33 and LSM4. Whole-cell lysates were prepared from HCT116 cells treated with 10□M VP16 for 18h or left untreated. Lysates were subjected to immunoprecipitation using RBM42 antibody or beads-only and subjected to immunoblot analysis with the indicated antibodies. Source data are provided as a Source Data file.

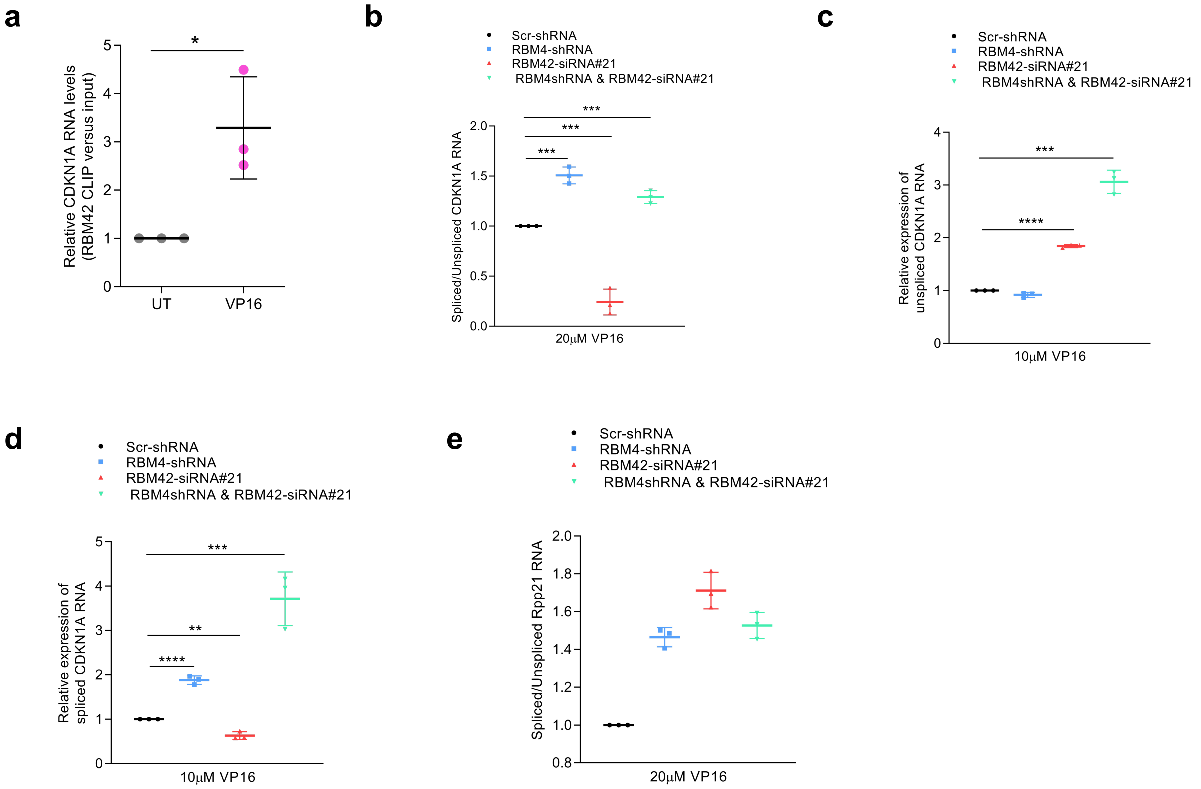

Supplementary Figure 7. Related to Figure 5. **(a)** RBM42 CLIP-qPCR shows induced binding of RBM42 to CDKN1A RNA following DNA damage. P-*value* = 0.02 **(b)** Co-depletion of RBM4 and RBM42 restores CDKN1A splicing integrity. RT-qPCR, as in Fig. 5d, except of using 20□M VP16. P-*value*(RBM4-sh)=0.0005; P-*value*(si#21)=0.0005; P-*value*(si#21 and RBM4-sh)=0.001 **(c-d)** RT-qPCR analysis showing the levels of unspliced (c) and spliced (d) CDKN1A transcripts normalized to GAPDH. (c) P-*value*(si#21)=0.0000001; P-*value*(si#21 and RBM4-sh)=0.00008. (d) P-*value*(RBM4-sh)=0.00008; P-*value*(si#21)=0.002; P-*value*(si#21 and RBM4-sh)=0.001.**(e)** Co-depletion of RBM4 and RBM42 doesn't affect Rpp21 splicing. RT-qPCR, as in Fig. 5d, shows the splicing efficiency of Rpp21 in control and HCT116 cells depleted either of RBM42, RBM4, or co-depleted of RBM42 and RBM4. Data are presented as mean ± s.d. (n=3 biologically independent experiments). The statistical tests are two-tailed, tow-sided *t-test*. *p < 0.05, **p < 0.01, ***p < 0.001, ****p < 0.0001. Source data are provided as a Source Data file.

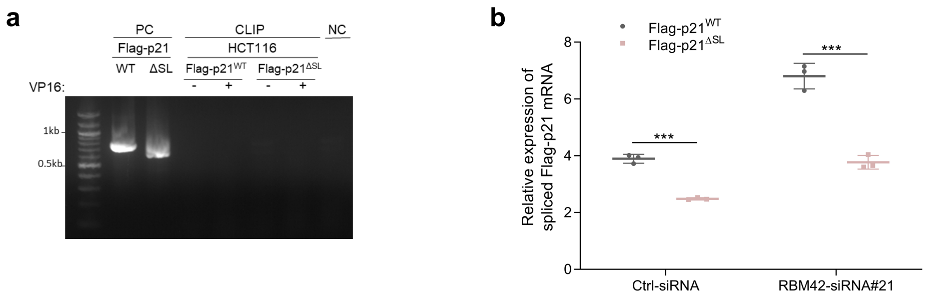

Supplementary Figure 8. Related to Figure 7. **(a)** PCR analysis showing lack of DNA contamination in the RNA samples (used for CLIP-pPCR in Figure 7d-e) prepared from HCT116 cells transfected with Flag-p21^{WT} and Flag-p21$^{\Delta SL}$ plasmids. PCR was performed using CMV-Fwd and CDKN1A-R495 primers. For a positive control (PC), PCR was performed using Flag-p21^{WT} and Flag-p21$^{\Delta SL}$ plasmids. For a negative control (NC), PCR was performed using a sample lacking plasmid DNA. **(b)** Quantification of RNA transcripts derived from Flag-p21^{WT} and Flag-p21$^{\Delta SL}$ plasmids transfected in HCT116 cells (related to Figure 7f). RNA samples, prepared from control (Ctrl) and RBM42 depleted HCT116 cells treated with 10µM VP16 for 18h, were subjected to RT-qPCR analysis using FFwd and FRev primers. Source data are provided as a Source Data file. P-*value*(Ctrl-siRNA) = 0.0002; P-*value*(RBM42-siRNA) = 0.0005. Data are presented as mean ± s.d. (n=3 biologically independent experiments). The statistical tests are two-tailed, tow-sided *t-test*. *p < 0.05, **p < 0.01, ***p < 0.001, ****p < 0.0001. Source data are provided as a Source Data file.
