# Supplementary material for: A dual role of RBM42 in modulating splicing and translation of CDKN1A/p21 during DNA damage response
Source: Nat Commun. 2023 Nov 22;14:7628. doi: 10.1038/s41467-023-43495-6 (PMC10665399; doi:10.1038/s41467-023-43495-6)
Supplement: Supplementary file 2 — Description of Additional Supplementary Files [file 41467_2023_43495_MOESM2_ESM.pdf]

Description of Additional Supplementary Files

File name: Supplementary Data 1
Description: RBM42 siRNA differential gene expression in untreated and VP16-treated HCT116 cells

File name: Supplementary Data 2
Description: RMATS alternative splicing analysis results - untreated cells

File name: Supplementary Data 3
Description: RMATS alternative splicing analysis results - VP16-treated cells

File name: Supplementary Data 4
Description: Differential exon expression results

File name: Supplementary Data 5
Description: Mass spec results of APEX-RBM42 interactome

File name: Supplementary Data 6
Description: RBM42 eCLIP results

File name: Supplementary Data 7
Description: list of commercial plasmids, plasmids generated in this study and the primers used for cloning.

File name: Supplementary Data 8
Description: list of primers used for PCR and qRT-PCR.

File name: Supplementary Data 9
Description: list of antibodies, chemicals and Critical Commercial Assays used in this study.
