# Supplementary material for: A dual role of RBM42 in modulating splicing and translation of CDKN1A/p21 during DNA damage response
Source: Nat Commun. 2023 Nov 22;14:7628. doi: 10.1038/s41467-023-43495-6 (PMC10665399; doi:10.1038/s41467-023-43495-6)
Supplement: Supplementary file 12 — Reporting Summary [file 41467_2023_43495_MOESM12_ESM.pdf]

nature portfolio

Corresponding author(s): Nabieh Ayoub
Last updated by author(s): Nov 2, 2023

# Reporting Summary

Nature Portfolio wishes to improve the reproducibility of the work that we publish. This form provides structure for consistency and transparency in reporting. For further information on Nature Portfolio policies, see our Editorial Policies and the Editorial Policy Checklist.

## Statistics

For all statistical analyses, confirm that the following items are present in the figure legend, table legend, main text, or Methods section.

| n/a | Confirmed | |
|---|---|---|
| ☐ | ☒ | The exact sample size (*n*) for each experimental group/condition, given as a discrete number and unit of measurement |
| ☐ | ☒ | A statement on whether measurements were taken from distinct samples or whether the same sample was measured repeatedly |
| ☐ | ☒ | The statistical test(s) used AND whether they are one- or two-sided<br>*Only common tests should be described solely by name; describe more complex techniques in the Methods section.* |
| ☒ | ☐ | A description of all covariates tested |
| ☐ | ☒ | A description of any assumptions or corrections, such as tests of normality and adjustment for multiple comparisons |
| ☐ | ☒ | A full description of the statistical parameters including central tendency (e.g. means) or other basic estimates (e.g. regression coefficient) AND variation (e.g. standard deviation) or associated estimates of uncertainty (e.g. confidence intervals) |
| ☐ | ☒ | For null hypothesis testing, the test statistic (e.g. $F$, $t$, $r$) with confidence intervals, effect sizes, degrees of freedom and $P$ value noted<br>*Give P values as exact values whenever suitable.* |
| ☒ | ☐ | For Bayesian analysis, information on the choice of priors and Markov chain Monte Carlo settings |
| ☒ | ☐ | For hierarchical and complex designs, identification of the appropriate level for tests and full reporting of outcomes |
| ☒ | ☐ | Estimates of effect sizes (e.g. Cohen's *d*, Pearson's *r*), indicating how they were calculated |

*Our web collection on statistics for biologists contains articles on many of the points above.*

## Software and code

Policy information about availability of computer code

| | |
|---|---|
| Data collection | ImageQuant LAS4000, BioRad GelDoc, ZEISS ZEN microscopy. |
| Data analysis | The following open-access software packages were used: ImageJ (1.8.0), Graphpad Prism (9.3.1), FastQC (0.11.9), Salmon (1.7.0), HISAT2 (2.1.0), RMATS (4.1.1), DESEQ2 (1.42), DEXSEQ (1.48), ShinyGO (0.741), and MaxQuant (2.2.0) |

For manuscripts utilizing custom algorithms or software that are central to the research but not yet described in published literature, software must be made available to editors and reviewers. We strongly encourage code deposition in a community repository (e.g. GitHub). See the Nature Portfolio guidelines for submitting code & software for further information.

## Data

Policy information about availability of data

All manuscripts must include a data availability statement. This statement should provide the following information, where applicable:

- Accession codes, unique identifiers, or web links for publicly available datasets
- A description of any restrictions on data availability
- For clinical datasets or third party data, please ensure that the statement adheres to our policy

Raw RNA-seq data was deposited at ArrayExpress with accession number E-MTAB-11877. Raw proteomic mass spec data are available via ProteomeXchange with identifier PXD034854. Raw eCLIP data was deposited at GEO with accession number GSE245744. All data supporting the findings of this study are available from the corresponding author upon request.

## Research involving human participants, their data, or biological material

Policy information about studies with human participants or human data. See also policy information about sex, gender (identity/presentation), and sexual orientation and race, ethnicity and racism.

| | |
|---|---|
| Reporting on sex and gender | N/A |
| Reporting on race, ethnicity, or other socially relevant groupings | N/A |
| Population characteristics | N/A |
| Recruitment | N/A |
| Ethics oversight | N/A |

Note that full information on the approval of the study protocol must also be provided in the manuscript.

# Field-specific reporting

Please select the one below that is the best fit for your research. If you are not sure, read the appropriate sections before making your selection.

☒ Life sciences ☐ Behavioural & social sciences ☐ Ecological, evolutionary & environmental sciences

For a reference copy of the document with all sections, see nature.com/documents/nr-reporting-summary-flat.pdf

# Life sciences study design

All studies must disclose on these points even when the disclosure is negative.

| | |
|---|---|
| Sample size | No sample size calculation was performed. The number of samples in each experiment is indicated in the figure legends and always n>=3 as is standard in the field. |
| Data exclusions | No data was excluded. |
| Replication | Results from all replicates were included in the results. The number of replicates is indicated in the figure legends and always n>=3. |
| Randomization | Sample allocation was performed randomly. |
| Blinding | Blinding was not needed as data is collected by unbiased software from at least 3 replicates which were performed independently. |

# Reporting for specific materials, systems and methods

We require information from authors about some types of materials, experimental systems and methods used in many studies. Here, indicate whether each material, system or method listed is relevant to your study. If you are not sure if a list item applies to your research, read the appropriate section before selecting a response.

### Materials & experimental systems

| n/a | Involved in the study | |
|---|---|---|
| ☐ | ☒ | Antibodies |
| ☐ | ☒ | Eukaryotic cell lines |
| ☒ | ☐ | Palaeontology and archaeology |
| ☒ | ☐ | Animals and other organisms |
| ☒ | ☐ | Clinical data |
| ☒ | ☐ | Dual use research of concern |
| ☒ | ☐ | Plants |

### Methods

| n/a | Involved in the study | |
|---|---|---|
| ☒ | ☐ | ChIP-seq |
| ☒ | ☐ | Flow cytometry |
| ☒ | ☐ | MRI-based neuroimaging |

## Antibodies

| | |
|---|---|
| Antibodies used | Goat monoclonal anti-RBM42 (WB 1:400) Santa Cruz Bio SC-248370<br>Rabbit monoclonal anti-RBM42 (WB 1:1000; IF1:300) Abcam Ab241504 |

| | |
|---|---|
| | Rabbit polyclonal anti-Histone H2A.X phospho Ser-139 Cell Signaling Technology 2577 (γH2AX) (WB 1:1000)<br>Mouse monoclonal anti-β-actin [AC-15] (WB 1:10,000) Sigma-Aldrich A5441<br>Mouse monoclonal anti-c-Myc [9E10] (WB 1:1000, ChIP 1μg) Santa Cruz Bio sc-40<br>Rabbit polyclonal anti-Histone H3 (WB 1:30,000) Abcam ab1791<br>Sheep anti-mouse IgG, HRP-linked (WB 1:10,000) Amersham NXA931<br>Goat Anti-Rabbit IgG (H+L) Peroxidase AffiniPure (WB 1:20,000) Jackson ImmunoResearch 111–035-003<br>Donkey anti-rabbit Alexa Fluor®488 (IF 1:500) Invitrogen A21206<br>Goat anti-rabbit IgG (H&L) Alexa Fluor® 647 (IF 1:300) Abcam ab150079<br>Streptavidin (HRP) (WB 1:5000) Abcam ab7403<br>Anti p21 (WB 1:2000) Abcam Ab227443<br>Anti p53 DO-1 (WB 1:1000, ChIP 1μg) Santa Cruz Bio SC-126<br>Anti Flag (WB: 1:5000, ChIP 1 μg, IF 1:500) Sigma F7425<br>Anti RBM4 (WB 1:200) Santa Cruz Bio SC-373852 |
| Validation | All primary antibodies used in this study were validated for specificity and reactivity according to the manufacturer's statements. Moreover, the specificity of RBM42 and RBM4 antibodies were confirmed using siRNA knockdown assays as appears in the manuscript. |

## Eukaryotic cell lines

Policy information about cell lines and Sex and Gender in Research

| | |
|---|---|
| Cell line source(s) | U2OS, HEK293T, and HCT116 cell lines were obtained from ATCC. |
| Authentication | None of the cell lines used were authenticated. |
| Mycoplasma contamination | Cell lines were not tested for mycoplasma contamination. |
| Commonly misidentified lines (See ICLAC register) | No misidentified cell lines were used. |

## Plants

| | |
|---|---|
| Seed stocks | N/A |
| Novel plant genotypes | N/A |
| Authentication | N/A |
