# Supplementary material for: A dual role of RBM42 in modulating splicing and translation of CDKN1A/p21 during DNA damage response
Source: Nat Commun. 2023 Nov 22;14:7628. doi: 10.1038/s41467-023-43495-6 (PMC10665399; doi:10.1038/s41467-023-43495-6)

Figure 1a

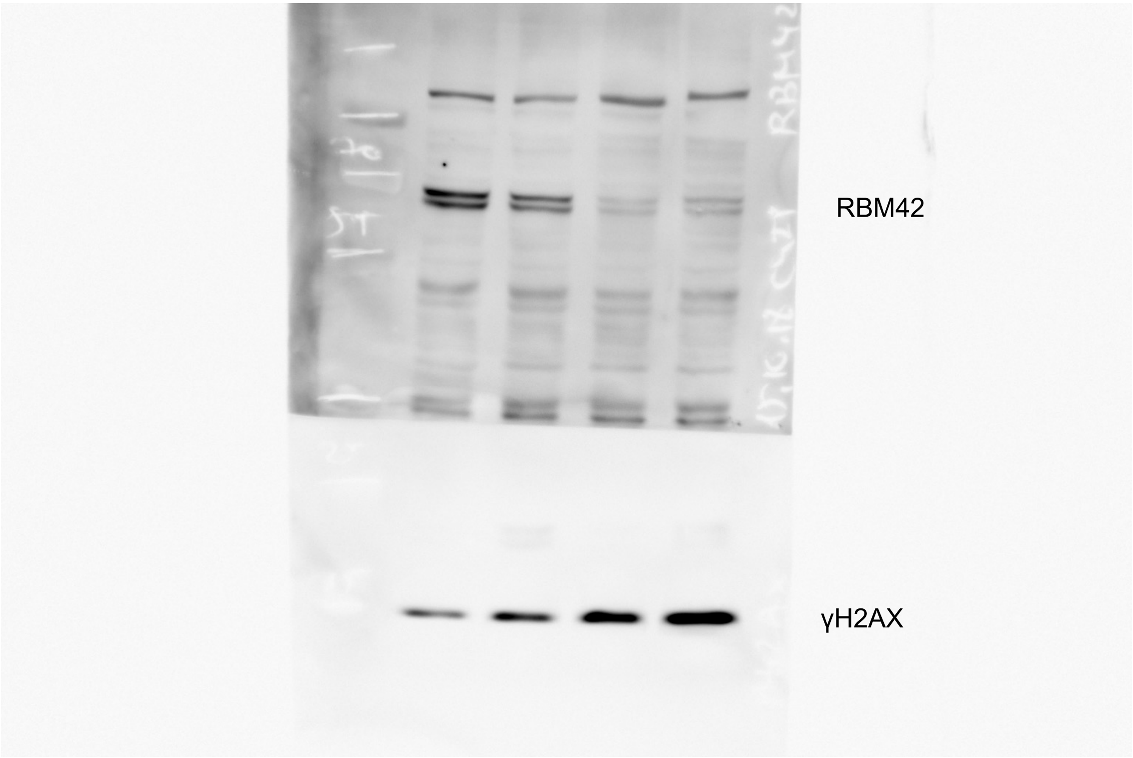

Figure 2a

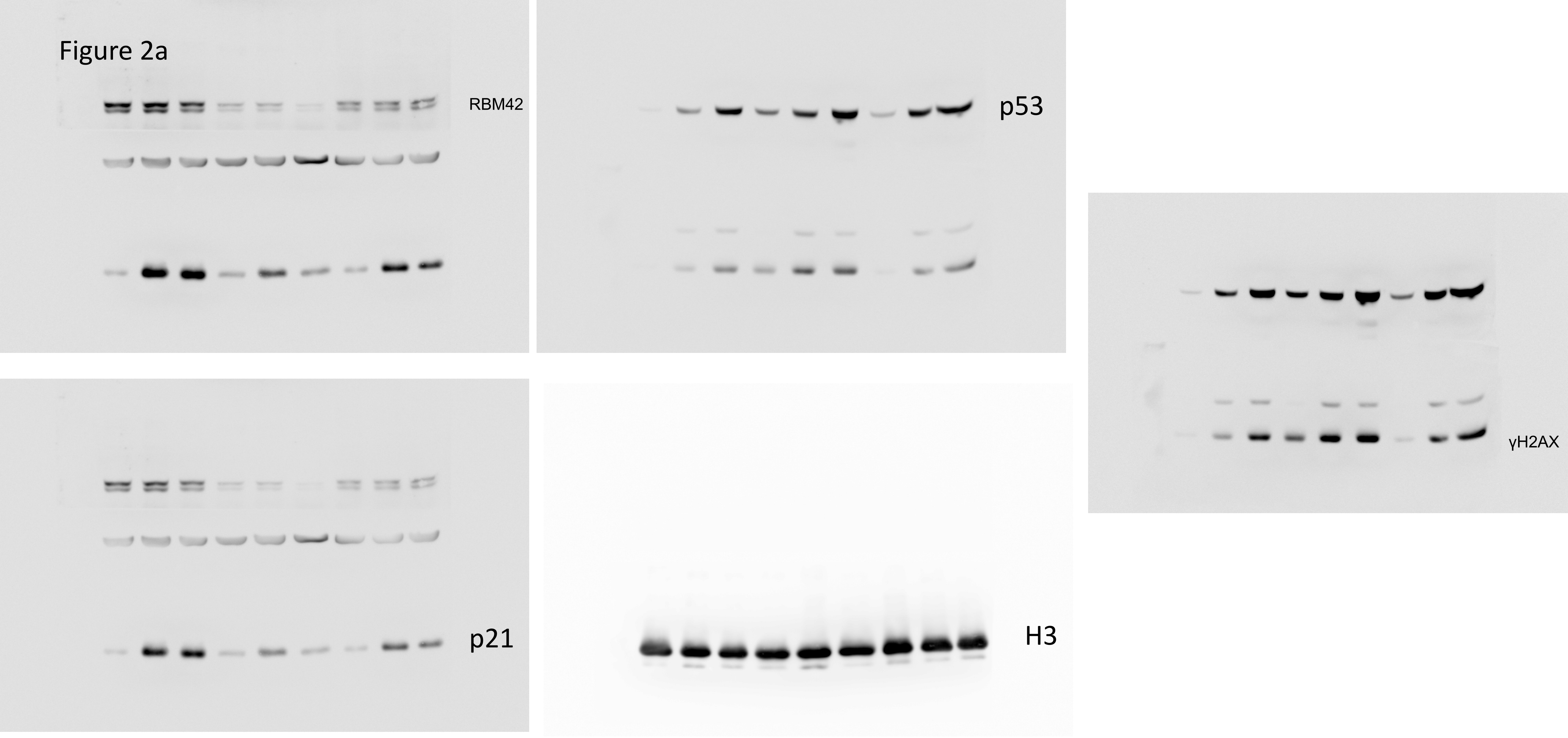

Figure 3h

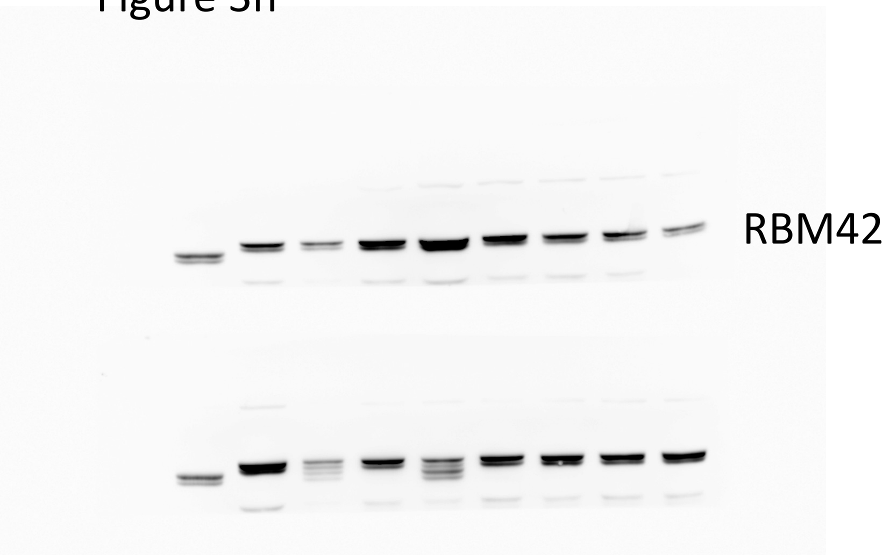

RBM42

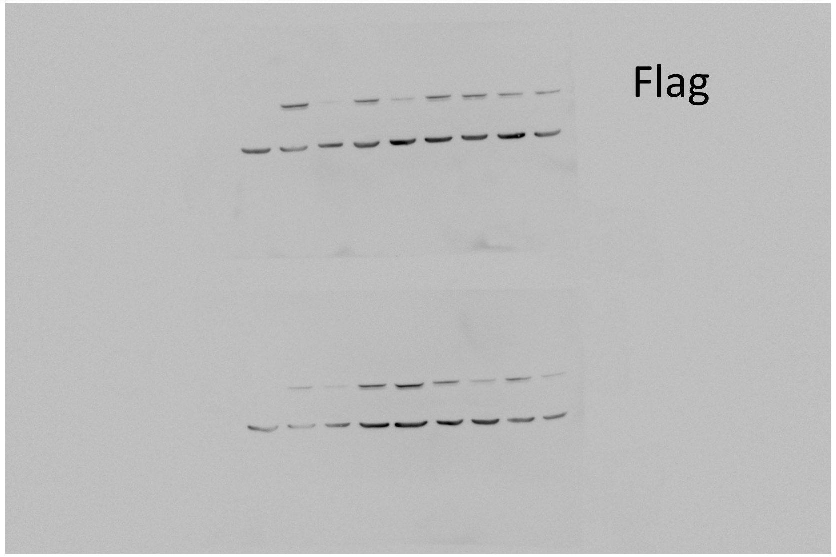

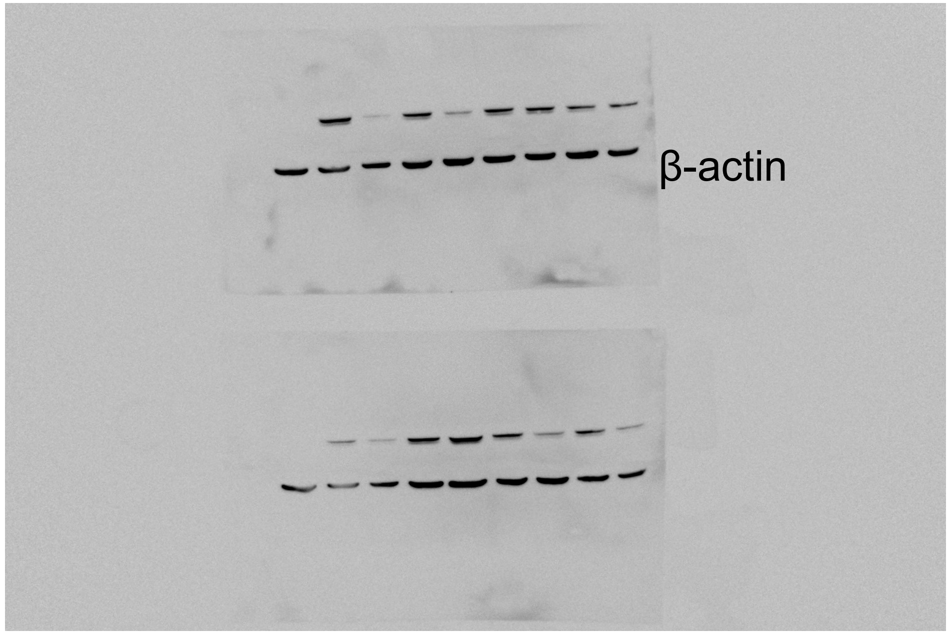

Figure 4a

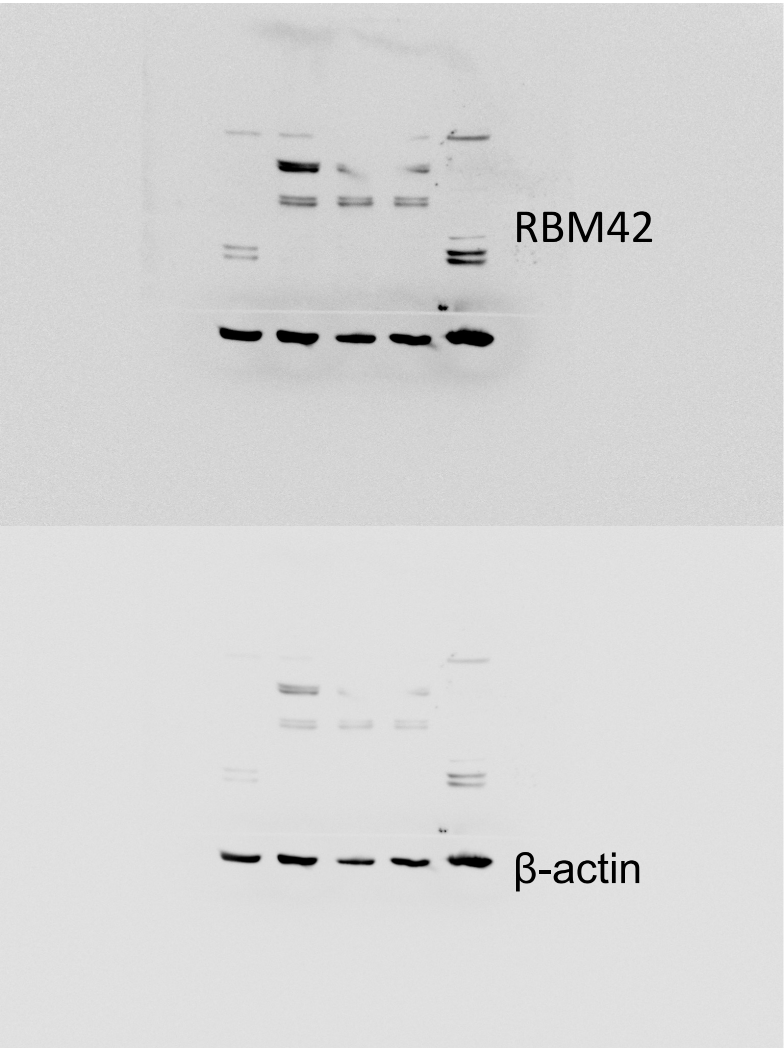

Figure 4c

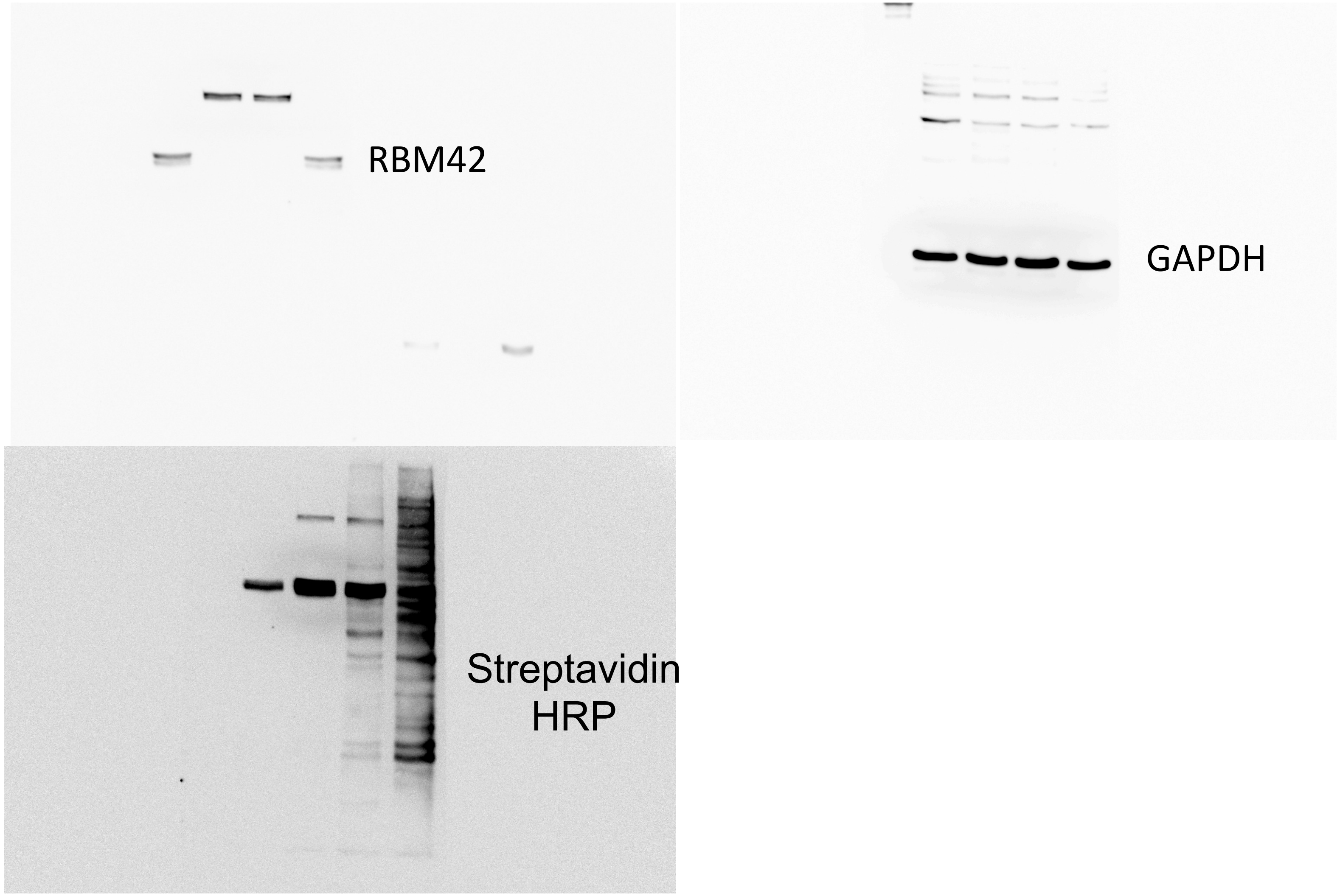

Figure 5a

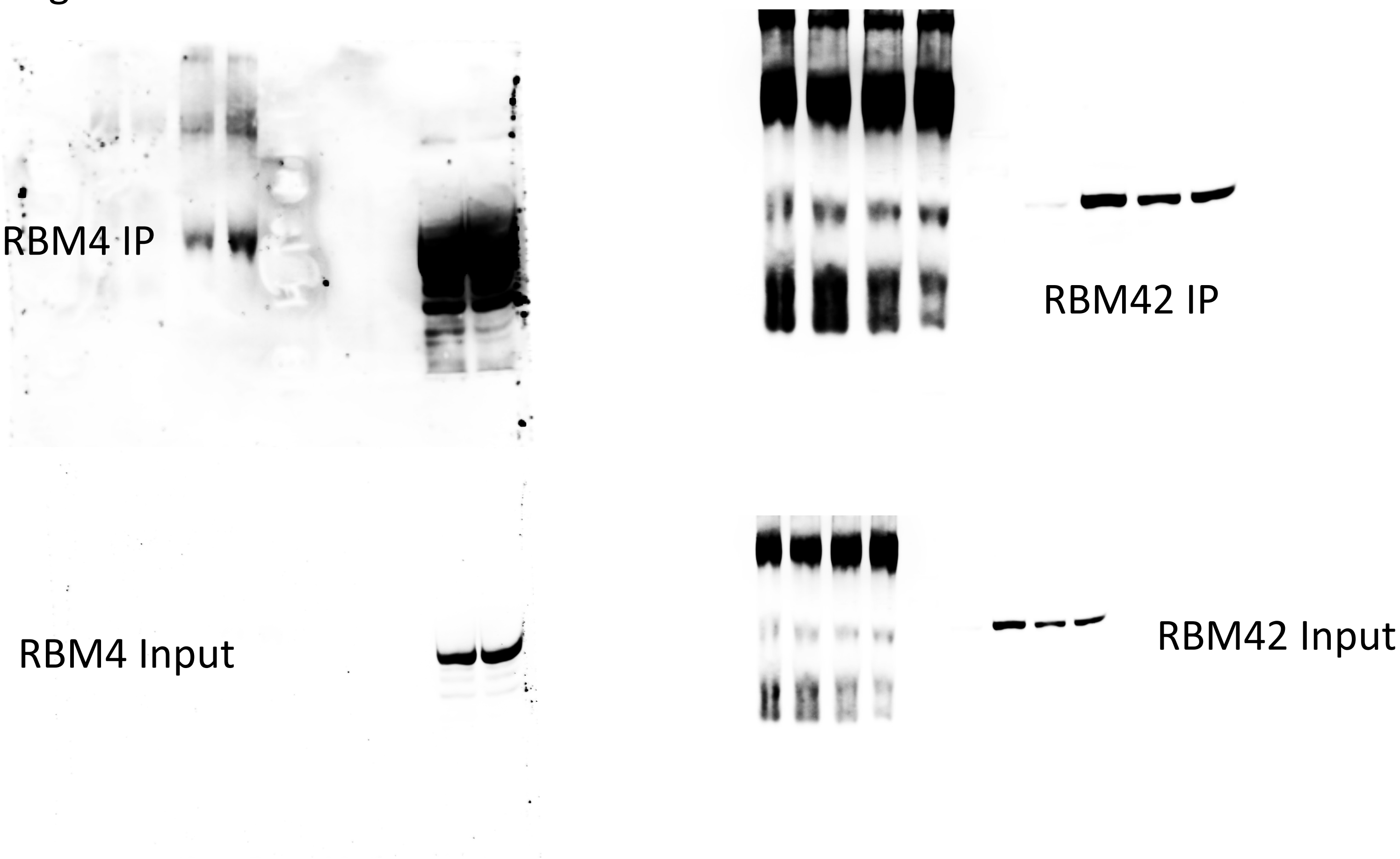

Figure 5f

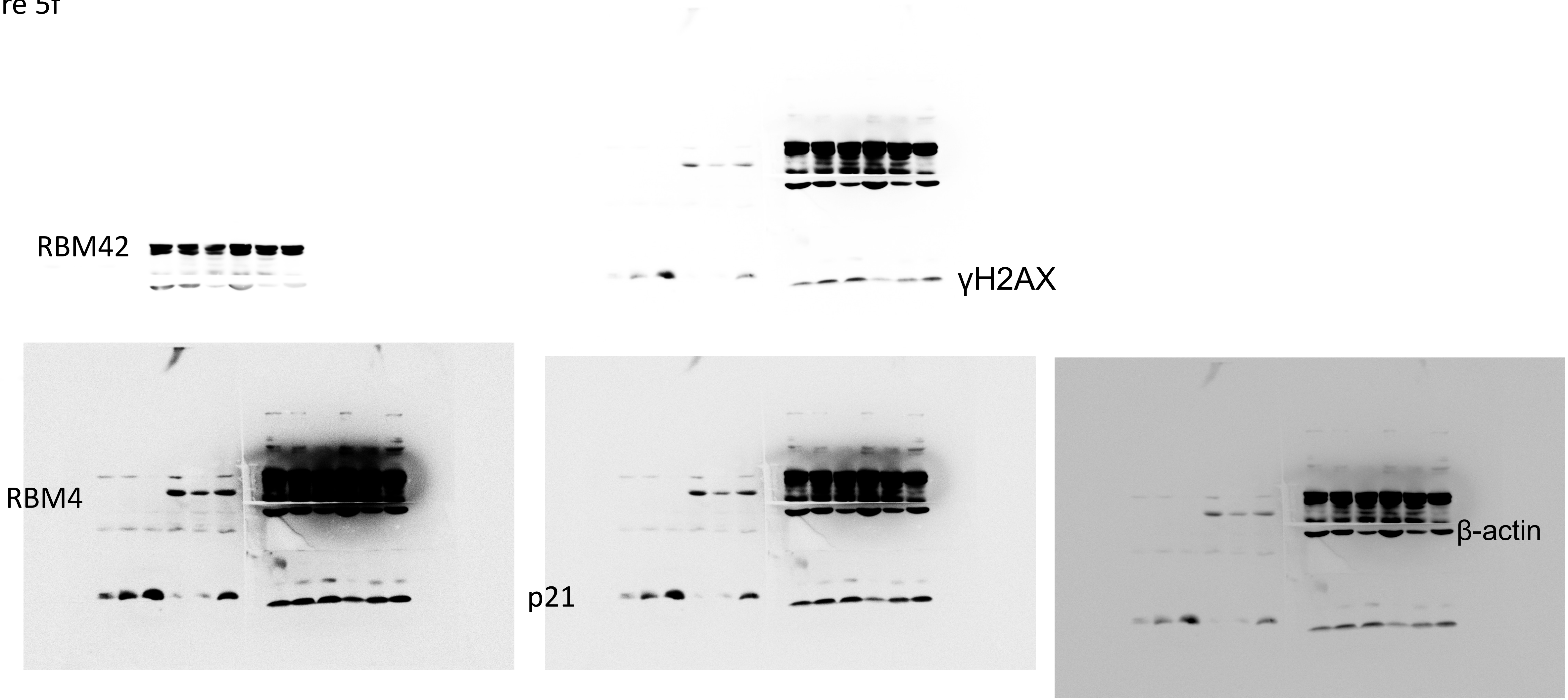

Figure 5g

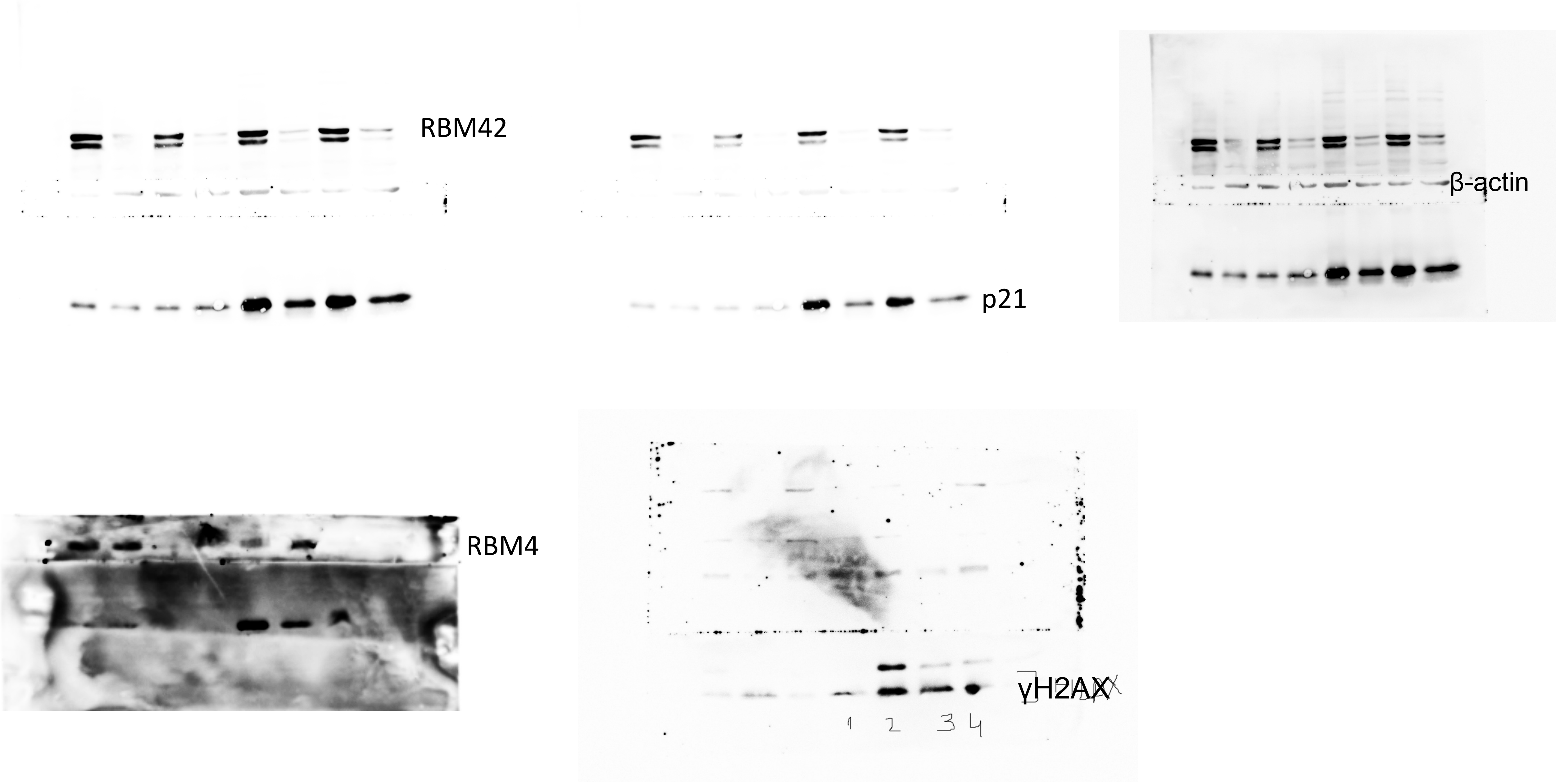

Figure 7a

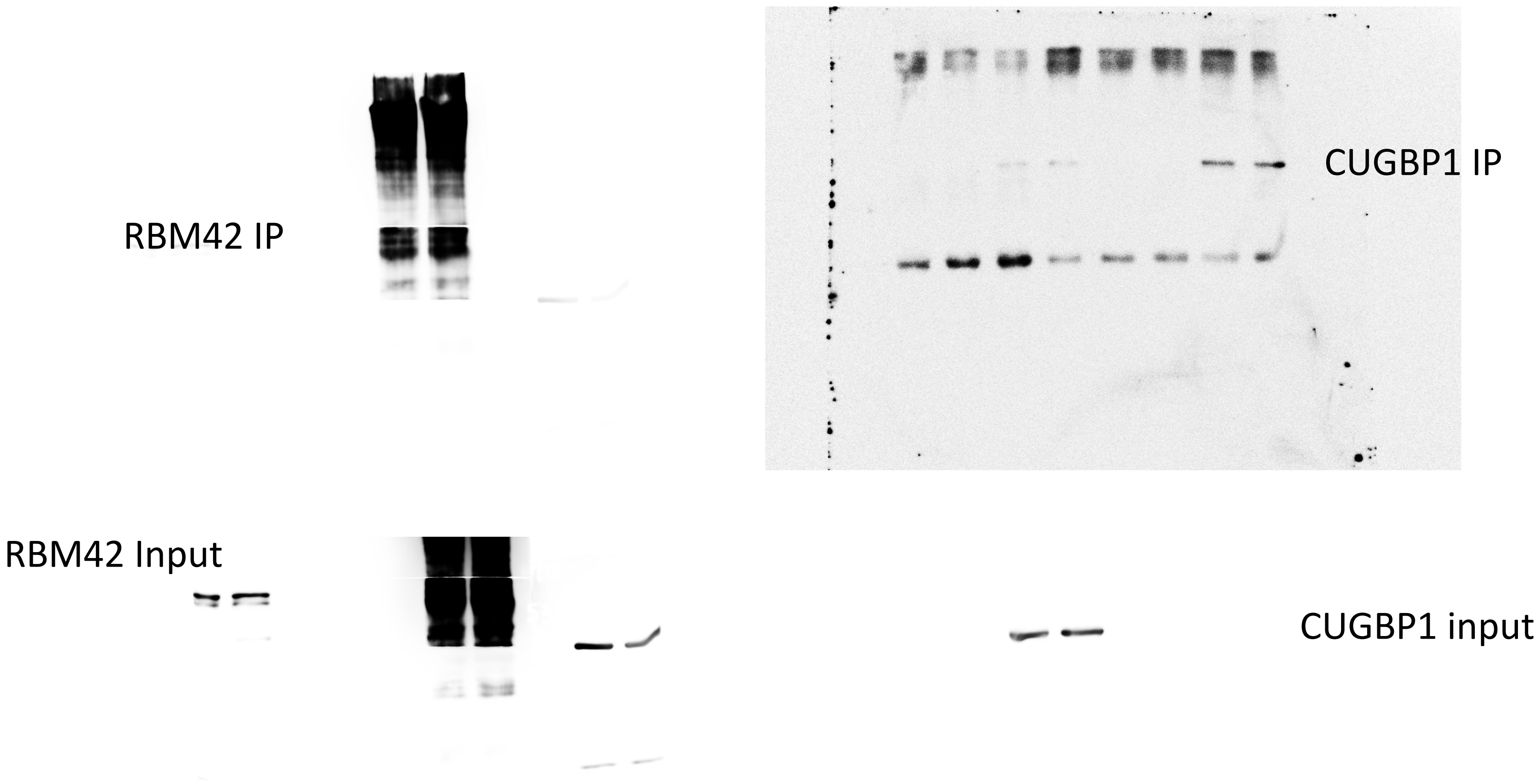

Figure 7f

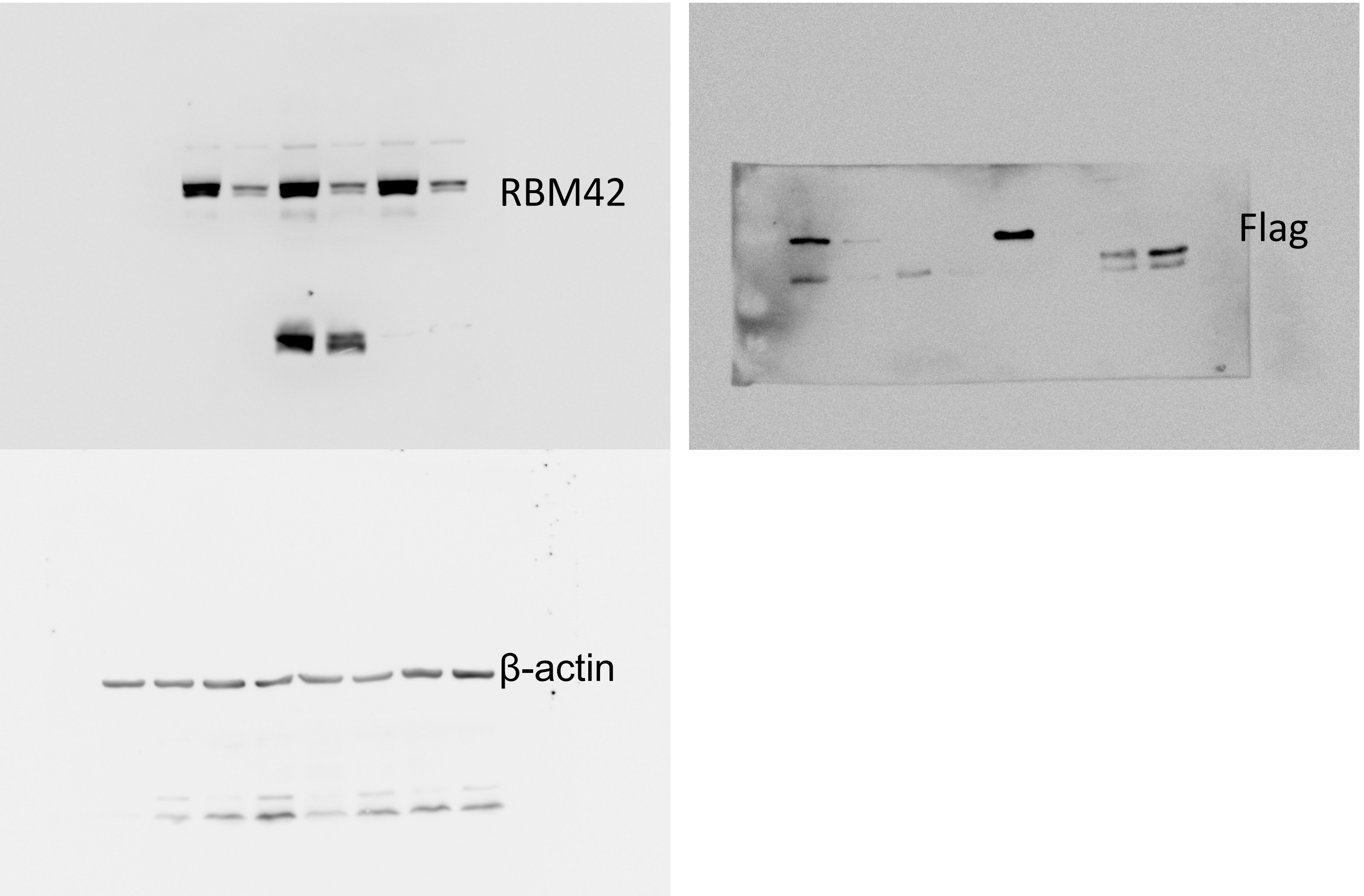

Supplement: Supplementary file 13 — Source Data [file 41467_2023_43495_MOESM13_ESM.zip › original_images_main_figures.pdf]
