# Supplementary material for: A dual role of RBM42 in modulating splicing and translation of CDKN1A/p21 during DNA damage response
Source: Nat Commun. 2023 Nov 22;14:7628. doi: 10.1038/s41467-023-43495-6 (PMC10665399; doi:10.1038/s41467-023-43495-6)

Supplementary figure 1a

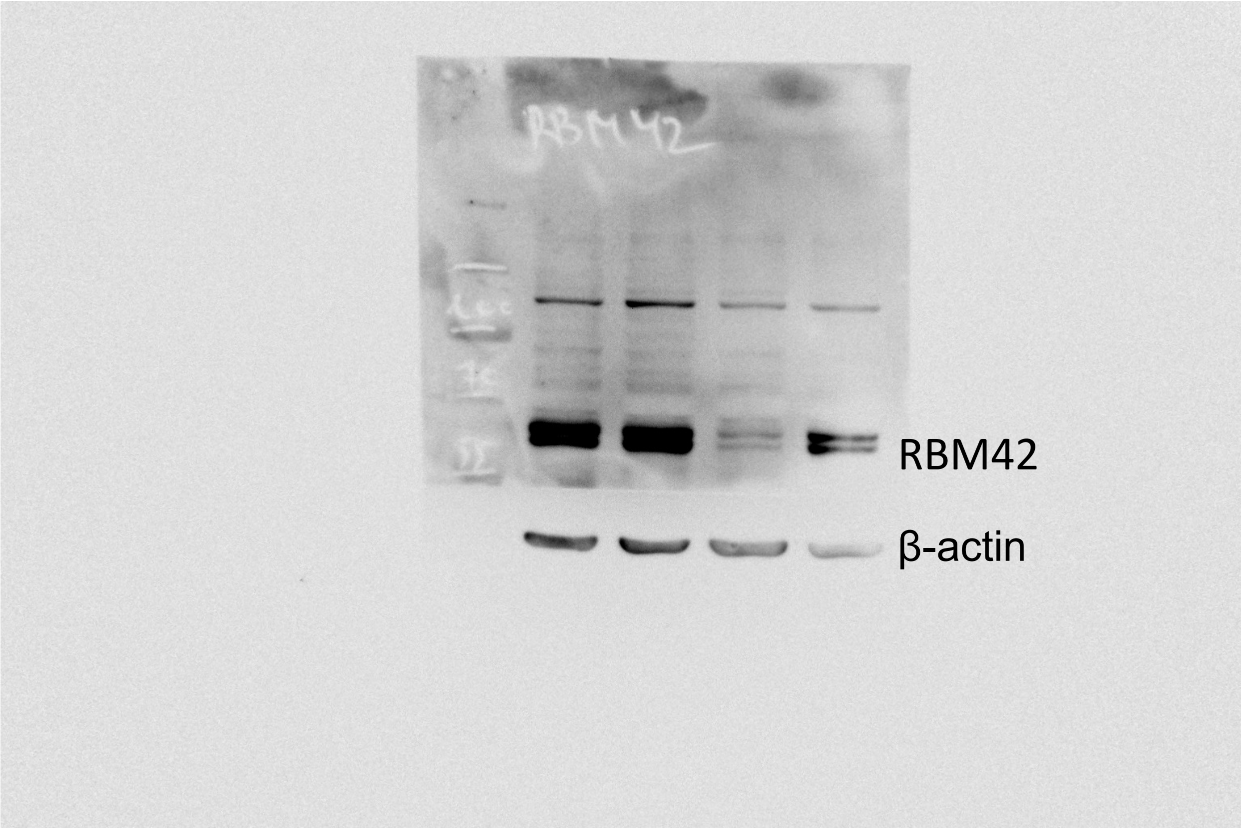

Supplementary figure 1b

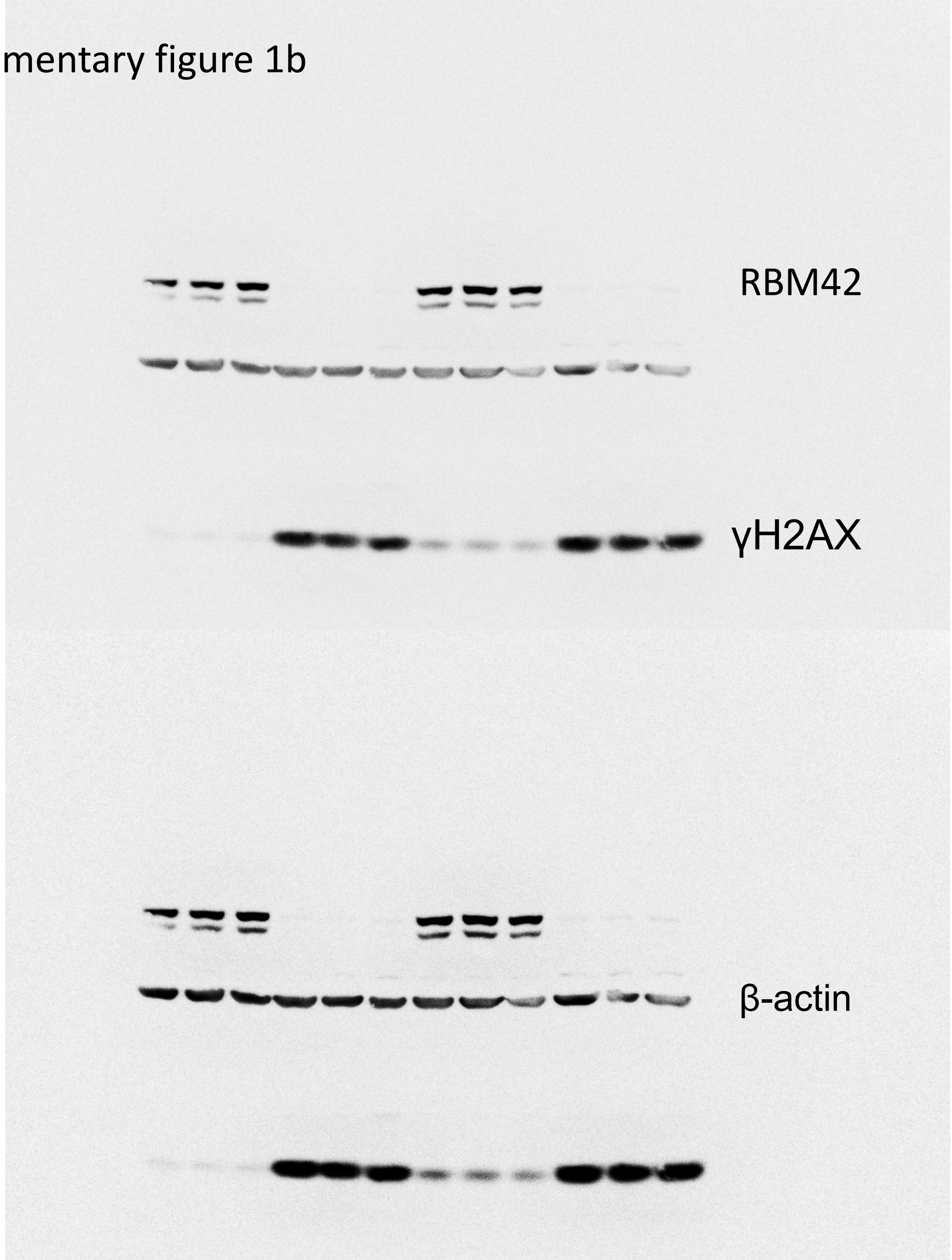

Supplementary figure 2a

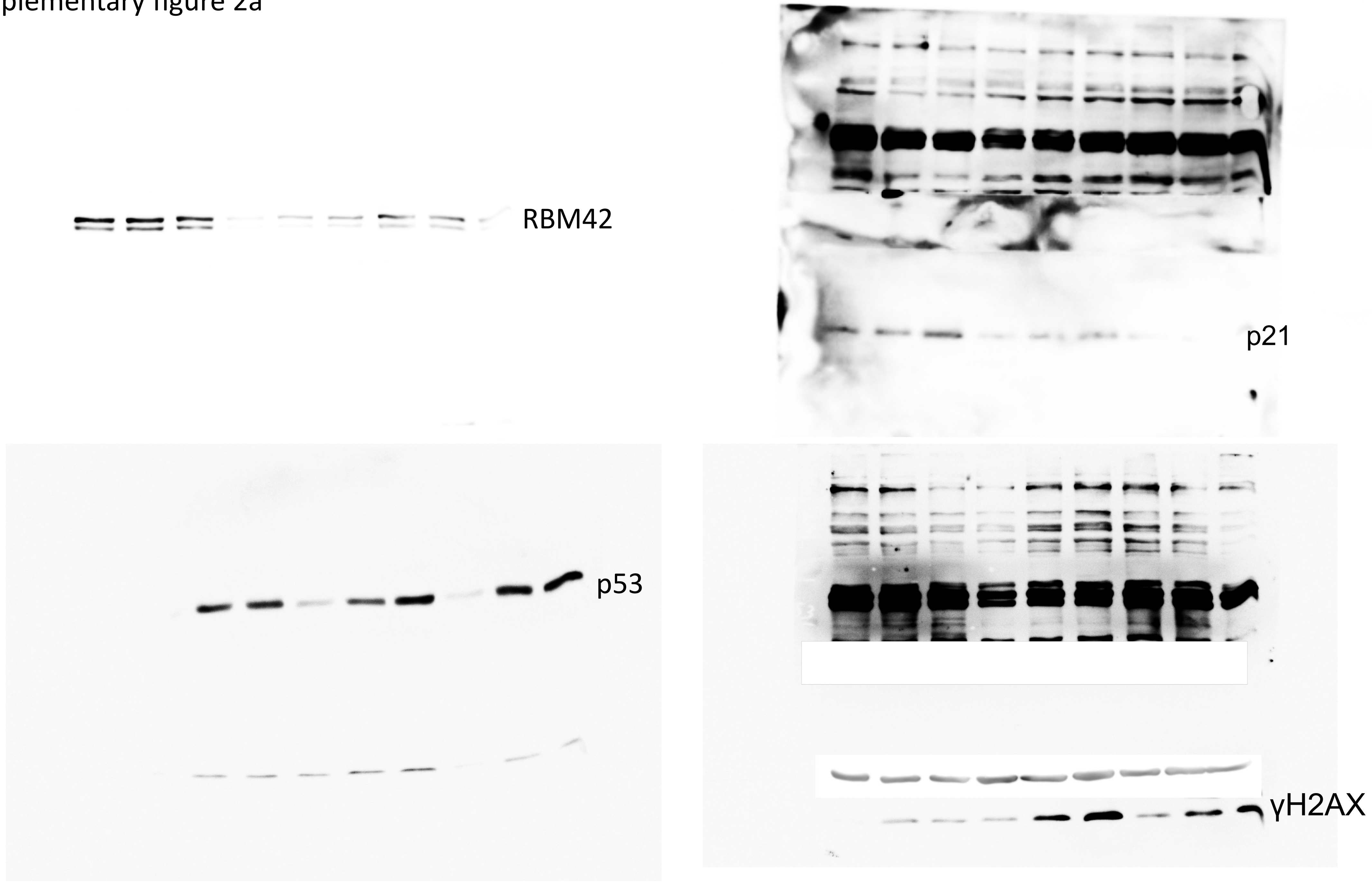

Supplementary figure 2a

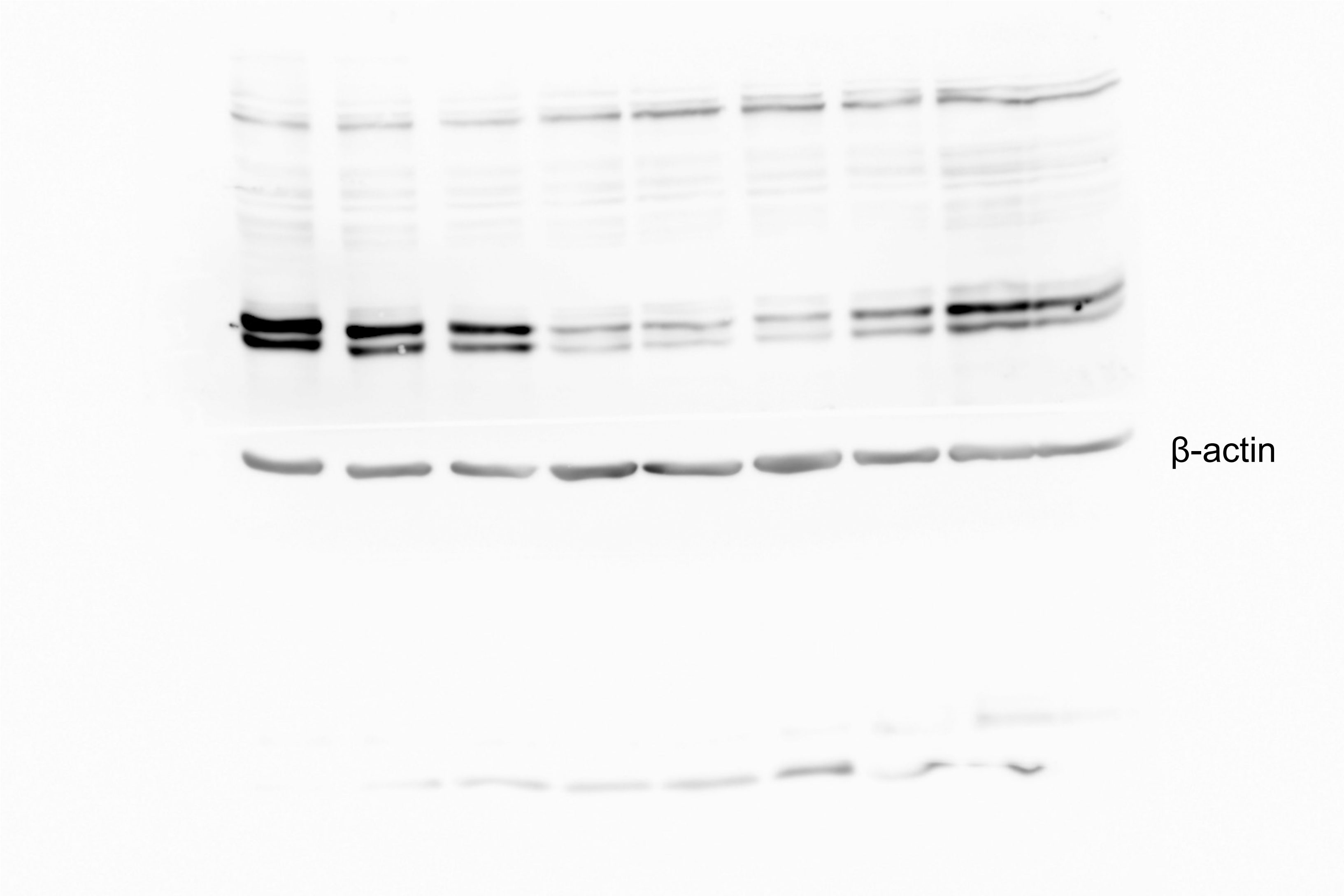

Supplementary figure 2b

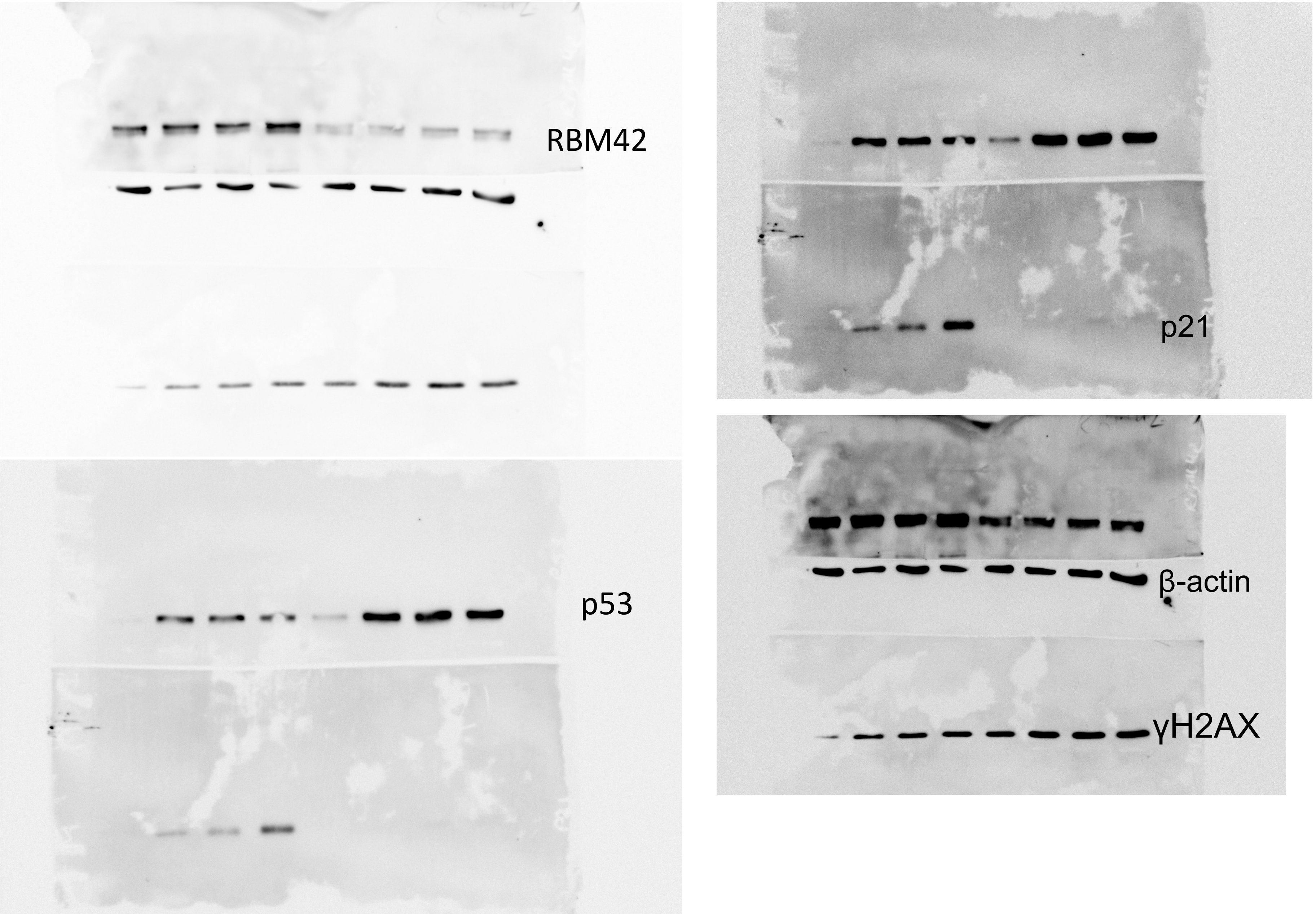

Supplementary figure 4h

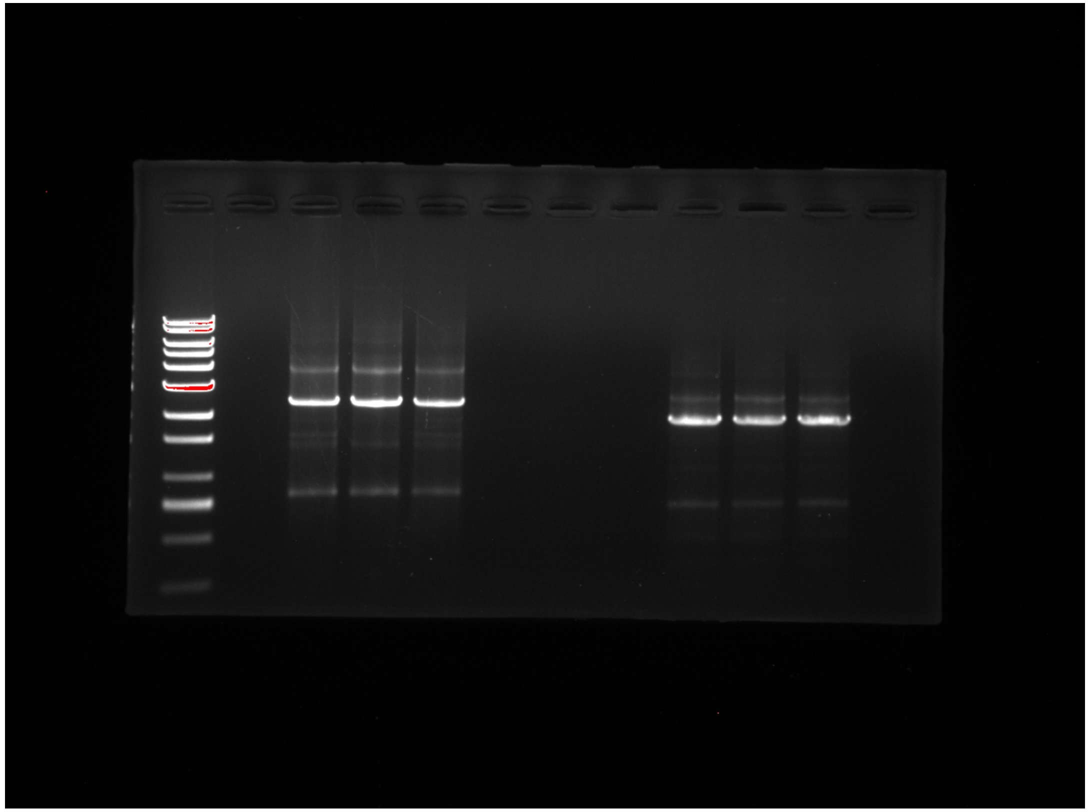

Supplementary figure 8a

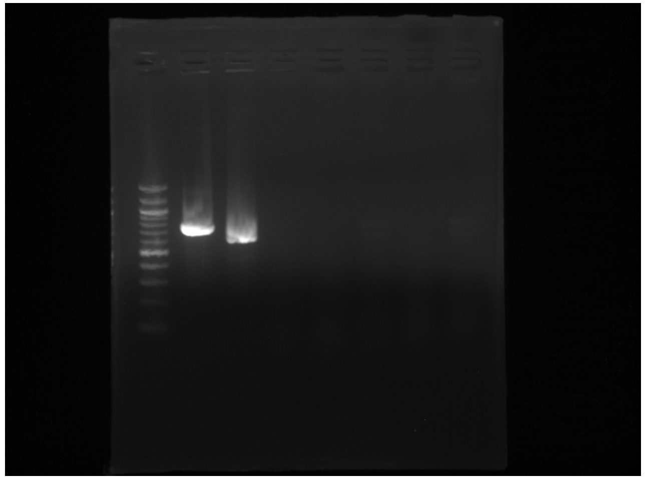

Supplement: Supplementary file 13 — Source Data [file 41467_2023_43495_MOESM13_ESM.zip › original_images_Supplementary_Information.pdf]
